# The Ciliogenic Transcription Factor RFX3 Regulates Early Midline Distribution of Guidepost Neurons Required for Corpus Callosum Development

**DOI:** 10.1371/journal.pgen.1002606

**Published:** 2012-03-29

**Authors:** Carine Benadiba, Dario Magnani, Mathieu Niquille, Laurette Morlé, Delphine Valloton, Homaira Nawabi, Aouatef Ait-Lounis, Belkacem Otsmane, Walter Reith, Thomas Theil, Jean-Pierre Hornung, Cécile Lebrand, Bénédicte Durand

**Affiliations:** 1 Département de Biologie Cellulaire et de Morphologie, University of Lausanne, Lausanne, Switzerland; 2 Centre de Génétique et de Physiologie Moléculaire et Cellulaire, CNRS UMR 5534, Université Claude Bernard Lyon 1, Lyon, France; 3 Centre for Integrative Physiology, University of Edinburgh, Edinburgh, United Kingdom; 4 Department of Pathology and Immunology, Faculty of Medicine, University of Geneva, Centre Médical Universitaire, Geneva, Switzerland; 5 National Center of Competence in Research Robotics, Ecole Polytechnique Fédérale, Lausanne, Switzerland; Université Catholique de Louvain, Belgium

## Abstract

The corpus callosum (CC) is the major commissure that bridges the cerebral hemispheres. Agenesis of the CC is associated with human ciliopathies, but the origin of this default is unclear. Regulatory Factor X3 (RFX3) is a transcription factor involved in the control of ciliogenesis, and *Rfx3*–deficient mice show several hallmarks of ciliopathies including left–right asymmetry defects and hydrocephalus. Here we show that *Rfx3*–deficient mice suffer from CC agenesis associated with a marked disorganisation of guidepost neurons required for axon pathfinding across the midline. Using transplantation assays, we demonstrate that abnormalities of the mutant midline region are primarily responsible for the CC malformation. Conditional genetic inactivation shows that RFX3 is not required in guidepost cells for proper CC formation, but is required before E12.5 for proper patterning of the cortical septal boundary and hence accurate distribution of guidepost neurons at later stages. We observe focused but consistent ectopic expression of *Fibroblast growth factor 8* (*Fgf8*) at the rostro commissural plate associated with a reduced ratio of GLIoma-associated oncogene family zinc finger 3 (GLI3) repressor to activator forms. We demonstrate on brain explant cultures that ectopic FGF8 reproduces the guidepost neuronal defects observed in *Rfx3* mutants. This study unravels a crucial role of RFX3 during early brain development by indirectly regulating GLI3 activity, which leads to FGF8 upregulation and ultimately to disturbed distribution of guidepost neurons required for CC morphogenesis. Hence, the RFX3 mutant mouse model brings novel understandings of the mechanisms that underlie CC agenesis in ciliopathies.

## Author Summary

The Corpus Callosum is the major brain commissure that links the two cerebral hemispheres in mammals. Absence or reduction of the corpus callosum is the most frequent brain malformation observed at birth in humans and leads to cognitive and behavioural deficits. Agenesis of the Corpus Callosum is frequently observed in ciliopathies, a group of human diseases due to defects in cilia assembly or function. However, the cellular origin of this brain malformation in these syndromes remains elusive. RFX3 transcription factor is a key regulator of ciliogenesis in mouse. Here, we show that the *Rfx3* mutant mouse shows impaired Corpus Callosum formation. By transplantation experiments, we demonstrate that this is due to defective distribution between the two hemispheres of a transient neuronal population (guidepost neurons) required for routing callosal axons. We show that this abnormal distribution is due to altered FGF8 signalling at early stages of brain development. Our observations show that small but focused signalling defects resulting in specific alterations in the distribution of guidepost neurons are responsible for corpus callosum agenesis.

## Introduction

The Corpus Callosum (CC), the major commissure of the brain, is composed of millions of axons that connect the two brain hemispheres [1], [2]. Malformation of the CC is one of the most frequent brain anomalies found at birth, and may occur in as much as 7/1000 of the total newborn population. The most severe form of CC malformation is its complete absence also called callosal agenesis.

In mouse, callosal axons first start to cross the midline during late gestation at E16.5 [3], [4]. Callosal axons are directed through the Cortical Septal Boundary (CSB) by several guidepost cell populations expressing guidance cues. Glial cell populations were first described to be involved in CC formation in this region [1], . Guidepost glial cells are found at the glial wedge (GW) of the lateral ventricles (initially described as the cortical septal plate [5]), in the *induseum griseum* (IG) of the medial pallium and in the so-called sling at the CSB [5]–[10]. More recently, GABAergic (γ-aminobutyric acidergic) neurons and glutamatergic neurons that populate transiently the CSB have also been shown to be involved in guiding callosal axons at the midline [11]. These glial and neuronal guidepost populations are also observed in the human foetal CC [2], [12].

In humans, malformations of the CC have been found to be associated with a variety of syndromes [13], [14]. In particular, a reduction or a complete absence of the CC has been found to be associated with several human syndromes recently recognized as ciliopathies [15], [16]. However, it is not known where and at what stage of embryonic development cilia are required for proper CC formation. Several mouse models defective in cilia formation or function have been described in the literature, but only few have been shown to be associated with CC malformations and none of them has so far been used to explore the molecular mechanisms that underlie CC development. One reason is that most mouse mutants for ciliary genes die early during embryogenesis and that the surviving mutants present severe brain malformations that preclude the study of late defects such as CC formation.

RFX transcription factors have been shown to play fundamental roles in the control of ciliogenesis by regulating many genes involved in cilia assembly or function [17]. *Rfx3* deficient mouse mutants exhibit several hallmarks of ciliopathies and in particular left-right asymmetry defects and hydrocephalus [18], [19]. We show here that *Rfx3* deficient mice also harbour marked defects in CC development leading in most cases to agenesis of the CC. RFX3 is first expressed throughout the anterior neural tube and is then progressively restricted to particular cell populations, particularly at the midline CSB, before and while pioneer callosal axons cross the midline. *Rfx3* loss of function leads to a distorted distribution of the neuronal but not of the glial guidepost cell populations that have both been shown to direct callosal axons through the midline. Reciprocal transplant experiments demonstrate that in *Rfx3−/−* brains, defects of the midline corticoseptal region are indeed responsible for improper crossing of the midline by callosal axons. However, conditional inactivation of *Rfx3* at specific time points in corticoseptal cell populations does not lead to CC defects, demonstrating that RFX3 is required early during brain development to pattern the CSB. We show that E12.5 *Rfx3* deficient brains present a mild expansion of *Fgf8* expression in the rostromedial septum, similar to a *Gli3* hypomorphic phenotype. We indeed show that GLI3 processing is altered in *Rfx3* deficient brains. Last we show, using organotypic slice cultures, that ectopic FGF8 expression disrupts guidepost neuronal distribution similar to the *in vivo* defects observed in *Rfx3* mutants. Altogether, our data show that loss of function of *Rfx3* at early stages of embryonic development is responsible for disturbed GLI3 processing and to small alterations in *Fgf8* expression, likely sufficient to induce dramatic aberrations in corticoseptal organization of guidepost neurons and consequently in CC formation. *Rfx3* mouse mutants thus appear to be particularly informative for understanding the molecular mechanisms that govern early midline patterning and offers a rare insight into the causes of CC defects in ciliopathies.

## Results

### *Rfx3* mutant mice are acallosal

**Figure 1:**
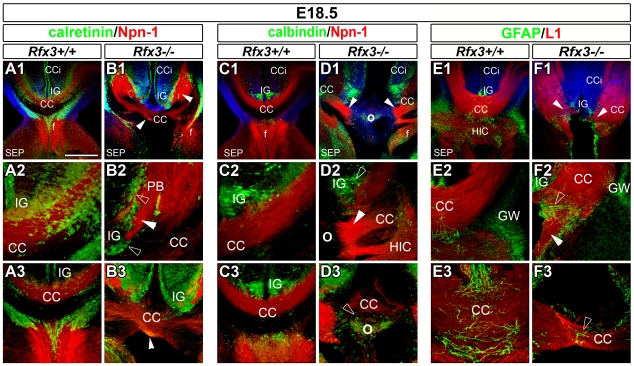
Abnormal callosal axon pathfinding in *Rfx3−/−* mice. (A–F) Immunohistochemistry for calretinin and Npn-1 (A1–A3 and B1–B3), for calbindin and Npn-1 (C1–C3 and D1–D3), and for GFAP and L1 (E1–E3 and F1–F3) in coronal CC sections from E18.5 WT (A1–A3, C1–C3 and E1–E3) or *Rfx3*−/− (B1–B3, D1–D3 and F1–F3) mice. A2, B2, C2, D2, E2 and F2 are higher magnifications of the lateral CC seen in A1, B1, C1, D1, E1 and F1, respectively. A3, B3, C3, D3, E3 and F3 are higher magnifications of the medial CC seen in A1, B1, C1, D1, E1 and F1, respectively. (A1–A3, C1–C3 and E1–E3) At E18.5, the hemispheres of WT brains have fused. Callosal fibres (in red) cross the midline and project into the contralateral cortex. (B1–B3, D1–D3 and F1–F3) Aberrant callosal axon bundles are observed in *Rfx3−/−* embryos (arrowheads). (B1–B3 and F1–F3) While the hemispheres have fused, most of callosal fibres do not cross the midline and form large ectopic bundles on the CC border, reminiscent of Probst bundles (PB). (D1–D3) Some *Rfx3−/−* embryos exhibit a more severe phenotype with an absence of midline hemispheric fusion and absolutely no callosal axons crossing the midline. In this case, a large bulge is observed along the inter-hemispheric fissure at the location where the callosal axons approach the midline (O). In all mutants, axonal defects are accompanied by cellular mis-positioning through the CC and the IG (open arrowheads). While calretinin+ or calbindin+ neurons, as well as, GFAP+ glia, are still present in the CC and the IG of *Rfx3−/−* mice, there is a midline disorganization and a lateral shift of these cell populations. Bar = 435 µm in A1, B1, C1, D1, E1, F1; 220 µm in A3, B3, C3, D2, D3, E2, F2 and 110 µm in A2, B2, C2, E3, F3.

The consequence of *Rfx3* inactivation on CC development was analysed on sections stained with haematoxylin-eosin or immunostained for specific guidance markers of callosal axons: Neuropilin1 (Npn-1) and L1CAM cell adhesion protein (L1) (Figure 1 and Figure S1). At E18.5, callosal axons have crossed the CC midline in wild-type (WT) mice (Figure 1A, 1C, 1E and Figure S1A). In contrast, *Rfx3*−/− mice exhibited partial (n = 4/11) to complete agenesis (n = 4/11) of the CC, with few or no callosal axons crossing the midline (Figure 1 and Figure S1). Remaining *Rfx3*−/− mice did not exhibit any obvious callosal defects (n = 3/11). In *Rfx3*−/− brains, many callosal axons reached the midline but, instead of crossing it, accumulated on both sides of the midline and formed dense axonal bundles called Probst Bundles (PB) (arrowheads, Figure 1 and Figure S1). In some animals there was a relatively mild phenotype in which the two cerebral hemispheres fused correctly, and a few callosal axons still crossed the midline albeit with abnormal trajectories (Figure 1B, 1F and Figure S1B). The most severe phenotype that we observed was a complete agenesis of the CC with no callosal axons crossing the midline. In these *Rfx3*−/− mice, the two cerebral hemispheres did not fuse correctly and displayed a large bulge along the inter-hemispheric fissure where callosal axons approach the midline (Figure 1D and Figure S1C, symbol O). Additionally, we observed in these embryos strong defects in the formation of the hippocampal commissure but not of the anterior commissure (Figure 1, Figure S1 and not shown). Thus *Rfx3* contributes to the formation of the CC and the hippocampal commissure.

### Expression of RFX3 in glutamatergic neurons of the developing CC and cortex

**Figure 2:**
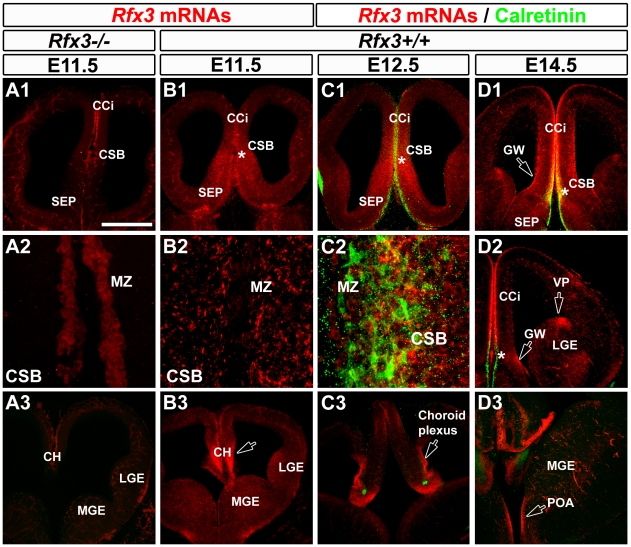
Expression pattern of *Rfx3* in the developing mouse telencephalon from E11.5 to E14.5. (A–D) *In situ* hybridization for *Rfx3* mRNAs on coronal brain sections of wild type (B1–B3) and *Rfx3−/−* (A1–A3) embryos at E11.5. *In situ* hybridizations for *Rfx3* (in red) combined with immunohistochemical staining for calretinin (C1–C3 and D1–D3) (in green) on coronal brain sections of wild type embryos at E12.5 (C1–C3) and E14.5 (D1–D3). A1, B1, C1 and D1 are coronal sections at the corticoseptal boundary (CSB, *) level, while A3, B3, C3 and D3 are caudal coronal sections at the level of the cortical hem (CH). A2, B2 and C2 are higher power views of the CSB seen in A1, B1 and C1 respectively. D2 is a lateral view of the telencephalon. (A and B) At E11.5, *Rfx3* is strongly expressed in wild type mice throughout the entire neuroepithelium of the CSB, and at more caudal levels in the cortical hem (B1–B3). The *Rfx3* hybridization signal is specific since no signal is visible in the same brain area of *Rfx3−/−* (A1–A3). (C–D) From E12.5 to E14.5, *Rfx3* expression is restricted to the cingulate cortex (CCi) that contains pioneer callosally projecting neurons, and throughout the CSB at the midline where the CC will form (C1–C2 to D1–D2). In addition, *Rfx3* is detected within the glial wedge (GW, open arrows) and the septum. (C3 to D3) On more caudal sections, *Rfx3* mRNAs are expressed in the cortical hem (CH), choroid plexus, ventral pallium (VP) and preoptic area (POA) (open arrows). Bar = 435 µm in A1, A3, B1, B3, C1, C3, D1, D2, D3 and 60 µm in A2, B2, C2.

To understand how RFX3 is involved in CC formation, we analysed *Rfx3* mRNA expression in coronal sections of the developing mouse telencephalon prior to and during CC formation. From E8 to E10.5, *Rfx3* was uniformly expressed in the entire neuroepithelium (not shown). From E11.5 to E16.5, *Rfx3* expression became progressively restricted to specific rostro-caudal levels in the telencephalon (Figure 2 and Figure S2). *Rfx3* hybridization signal was strong at the CSB (*) where the CC will form, and in the cingulate cortex (CCi) that contains pioneer callosal projection neurons [3], [4] (Figure 2B–2D). In addition, *Rfx3* was expressed in the primordium of the IG and the ventricular zone of the GW at the border of the lateral ventricles (Figure 2B1, 2C1, 2D1–D2). Both regions surround the CSB and are known to be important for CC formation [1], [7], [8]. High *Rfx3* expression was also observed at more rostral levels in the retrobulbar region and at caudal levels in the cortical hem (CH), the choroid plexus, the ventral pallium (VP) laterally, as well as, in the preoptic area (POA) (Figure 2B3, 2C3 and 2D2–D3, open arrows). From E16.5 to birth, *Rfx3* expression in the rostral telencephalon was restricted to the IG, the GW and the cerebral cortex (Figure S2E, S2G and Figure S3C, S3E to S3H).

To clarify the nature of the embryonic midline cells expressing RFX3, we performed co-labelling experiments with markers for different cell types. Glutamatergic guidepost neurons colonizing the forming CC express the calcium binding protein calretinin as well as several transcription factors known to promote the glutamatergic fate such as empty spiracles homolog 1 (EMX1) (Figure S3A) and T-box brain transcription factor 1 (TBR1) [11] (Figure S2 and Figure S3A). In the embryonic IG, part of the neurons express calbindin and are also glutamatergic since they express EMX1 (Figure S3B). In addition, GABAergic guidepost neurons can be identified using a GAD67-GFP mouse line in which the green fluorescent protein (GFP) is reliably expressed in GABAergic neurons. Finally, CC guidepost glia of the IG and of the GW can be distinguished by Nestin, Glutamate Aspartate Transporter (GLAST) and Glial Fibrillary Acidic Protein (GFAP) expression [5], [6].

At the CSB, *Rfx3* was expressed in glutamatergic guidepost neurons labelled for calretinin, reelin and TBR1 as early as E14.5 (Figure 2D and Figure S2A, S2B and S2D, *). In the IG, *Rfx3* mRNA was detected in glutamatergic neurons labelled for the calcium binding protein calbindin (Figure S2F). After E16.5, at the brain level where the cerebral hemispheres have already fused, *Rfx3* was no longer expressed by glutamatergic guidepost neurons of the CC white matter (Figure S2E1, arrow). *Rfx3* was still expressed by glutamatergic calretinin+ neurons of the marginal zone (MZ) and calbindin+ neurons of the IG (Figure S2E2 and S2G). We observed no co-localization between GAD67-GFP and *Rfx3* in neurons from E14.5 to E18.5 (not shown and Figure S3C). Thus, *Rfx3*-expressing neurons of the corticoseptal region are strictly glutamatergic.

Moreover, *Rfx3*-positive cells populating the ventricular zone in the GW region from E14.5 to E18.5 are radial glial cells, labelled for Nestin, GFAP and GLAST (Figure S3D–S3F). However, no such overlap can be observed in the IG region, confirming that *Rfx3*-positive cells are not glial cells in the IG (Figure S3F1).

In addition to *Rfx3* expression in the midline, we also observed at E12.5, that *Rfx3* mRNA was detected in pioneer calretinin+ glutamatergic cortical neurons of the preplate (Figure 2C). From E13.5 to E16.5, *Rfx3* mRNA was found in calretinin+ glutamatergic neurons in all layers of the developing cortex (Figure 2D and Figure S2B–S2E, S3E) and at E18.5 it was found in the projection neurons of the upper cortical layers labelled for Special-AT-rich sequence Binding protein 2 (SATB2) and in those of the lower cortical layers labelled for COUP-TF Interacting Protein 2 (CTIP2) (Figure S3G, S3H). By contrast, we never detected any *Rfx3* hybridization signal in the reelin+ Cajal Retzius cells or calbindin+ neurons of the cortical MZ (Figure S2A, S2F).

The presence of *Rfx3* transcripts at the midline in the corticoseptal region and in the cerebral cortex is consistent with the importance of this gene in CC formation. Given the large distribution of RFX3 in the embryonic brain, it might contribute to the proper development of the cortex or of midline structures. We, thus, examined if these different regions are affected by *Rfx*3 inactivation.

### RFX3 is not required in callosal axons for proper CC formation

We first analysed the cerebral cortex of *Rfx3* mutants. In *Rfx3−/−* cortex, the laminar distribution of SATB2+ and CUX1+ (Cut-like homeobox 1) callosally projecting neurons [20]–[26] was normal (Figure S4A–S4D). In addition, CTIP2+ cortical layer V and TBR1+ cortical layers V–VI which contain about 20% of the callosally projecting neurons, were similar in mutant and WT brains (Figure S4E–S4H).

To study if RFX3 expression in cortical neurons is necessary for axonal growth in the CC, we investigated whether the targeted inactivation of *Rfx3* in pyramidal cortical neurons results in pathfinding defects. Using a Ngn2-CreER driver line, we induced recombination of a *Rfx3* floxed allele in neurogenin 2 (NGN2)-derived glutamatergic projection neurons of the cortex by tamoxifen application at E13.5 [27], [28]. While *Rfx3* was not any more expressed in the cerebral cortex of *Rfx3^f/f^*; *Ngn2-CreERtm^+/−^* mice, (compare Figure S4I2 and S4J2), the SATB2+ callosally projecting neurons and the CC still formed normally (n = 7/7; Figure S4J1 and S4L). This result shows that the loss of *Rfx3* in cortical pyramidal neurons is not responsible for callosal axon guidance defects.

### RFX3 is required for proper distribution of guidepost neurons in the CSB

To determine if RFX3 was required for the development of the CSB we followed the organization of guidepost cells in mutant CSB compared to WT. We first followed the distribution of CC guidepost glutamatergic neurons in *Rfx3−/−* mice at E18.5, after callosal axons have crossed the midline. Glutamatergic neurons of the CC, labelled for TBR1 and calretinin were shifted laterally, leaving a large portion of the CC devoid of neurons (not shown and Figure 1A and 1B, open arrowheads). Calretinin+ and calbindin+ glutamatergic neurons were both severely disorganized through the IG (Figure 1A–1D, open arrowheads). In addition, a progressive disorganization of CC glial cells was noticed in *Rfx3−/−* CC regions. The GFAP-positive astrocyte-like cells of the IG and of the midline were disorganized and the curvature of the radial glial processes was increased (Figure 1E and 1F, open arrowheads). Because this disorganization could be a secondary effect of callosal misrouting, we also looked at the distribution of guidepost cells before callosal axons cross the midline.

**Figure 3:**
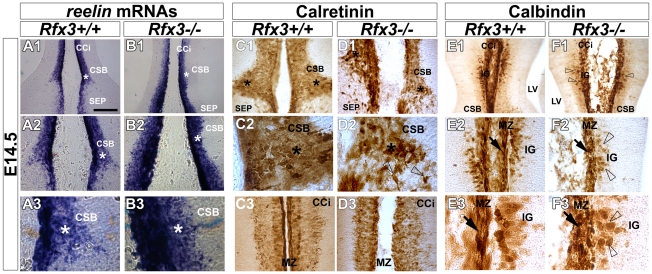
Aberrant localization of midline neurons before CC formation at E14.5. *In situ* hybridization for *reelin* mRNAs (A1–A3 and B1–B3) and DAB staining for calretinin (C1–C3 and D1–D3) and calbindin (E1–E3 and F1–F3) on coronal rostromedial slices from E14.5 WT (A1–A3, C1–C3 and E1–E3) and *Rfx3*−/− (B1–B3, D1–D3 and F1–F3) mice. A2, A3, B2, B3, C2 and D2 are higher power views of the corticoseptal boundary (CSB,*) seen in A1, B1, C1 and D1 respectively. E2, E3, F2 and F3 are higher power views of the induseum griseum (IG) region seen in E1 and F1, respectively. C3 and D3 are higher power views of the cortical marginal zone (MZ) seen in C1 and D1 respectively. (A–F) As early as E14.5, before inter-hemispheric midline fusion occurs, the organization of reelin+ (B1–B3), calretinin+ (D1–D2) and calbindin+ (F1–F2) midline neurons is severely affected in the CSB and the IG regions of the *Rfx3*−/− mutant (arrows and open arrowheads). In addition, the neurons lose their tangential organization through the *Rfx3*−/− cortical MZ (B3, D3 and F2–F3; arrows). Bar = 300 µm in A1, B1, E1, F1; 150 µm in A2, B2, C1, C3, D1, D3, E2, F2 and 60 µm in A3, B3, C2, D2, E3, F3.

**Figure 4:**
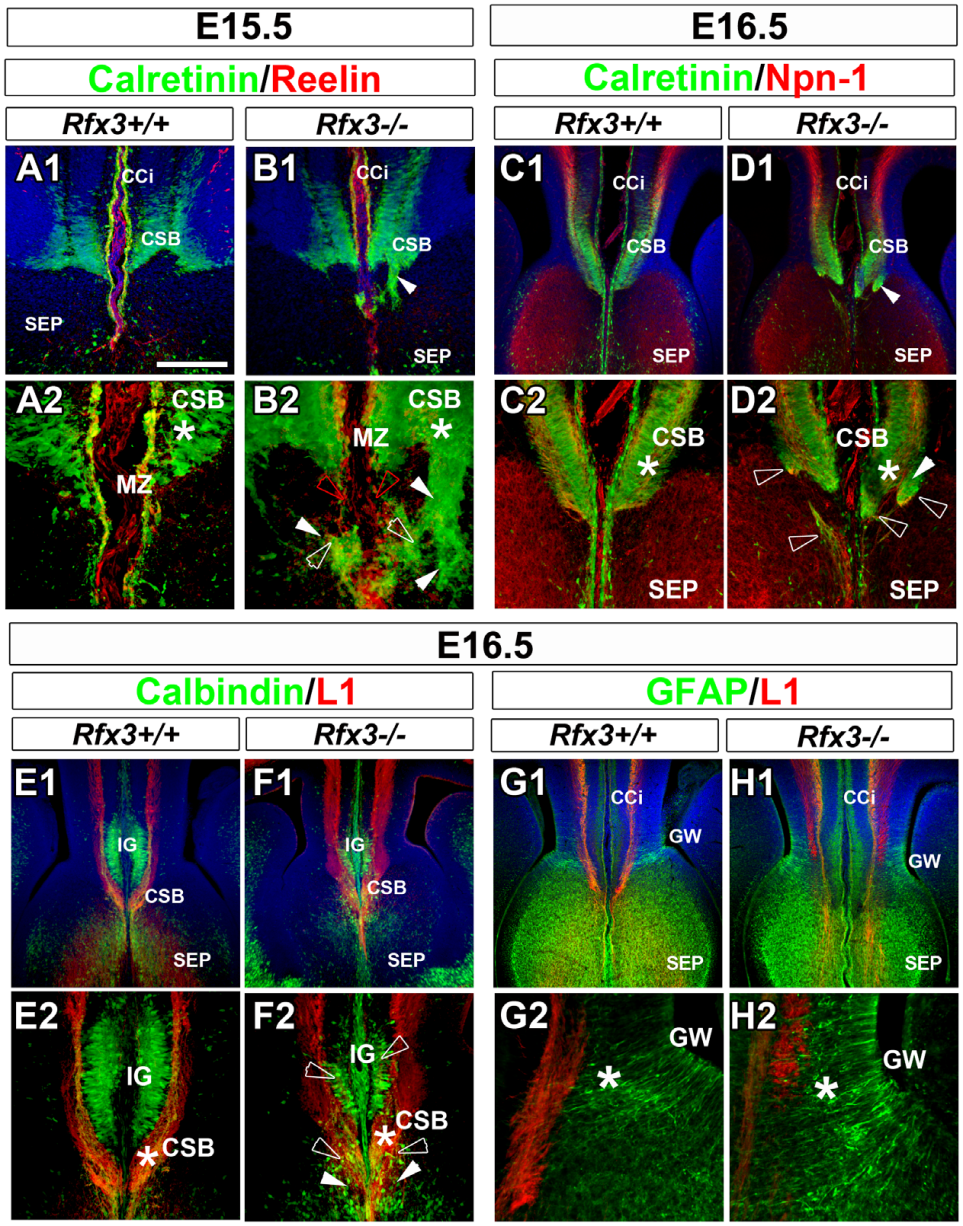
Abnormal neuron localization and aberrant callosal axon pathfinding at the onset of CC formation. (A–H) Immunohistochemistry for calretinin and reelin (A1–A2 and B1–B2), for calretinin and neuropilin-1 receptor (Npn-1) (C1–C2 and D1–D2), for calbindin and L1 receptor (E1–E2 and F1–F2) and for GFAP and L1 receptor (G1–G2 and H1–H2), in coronal CC sections from WT (A1–A2, C1–C2, E1–E2 and G1–G2) and *Rfx3*−/− (B1–B2, D1–D2, F1–F2 and H1–H2) mice. A2, B2, C2, D2, E2, F2, G2 and H2 are higher magnifications of the midline seen in A1, B1, C1, D1, E1, F1, G1 and H1. (A1–A2 to D1–D2) From E15.5 to E16.5, calretinin+ guidepost neurons fail to form a well organized band of neurons at the CSB (*) and are dispersed in the septum of *Rfx3−/−* mice (B2 and D2, white open arrowheads). Reelin+ and calretinin+ neurons loose their tangential organization through the cortical marginal zone (MZ) (compare B2 to A2, red open arrowheads). (E1–E2 and F1–F2) At E16.5, calbindin+ neurons (green) do not organize appropriately within the indusium griseum (IG) and accumulate at the CC midline in *Rfx3*−/− mice (compare F2 to E2, open arrowheads). (G1–G2 and H1–H2) At E16.5, the organization of GFAP+ glial cell populations within the CC is indistinguishable between WT and *Rfx3*−/− mice. (A to H) Axonal misrouting of pioneer callosal axons from E15.5 to E16.5. (A1–A2, C1–C2, and E1–E2) In WT brains, pioneer callosal fibres grow within the CSB and reach the midline. (B1–B2, D1–D2 and F1–F2) In *Rfx3−/−* brains, most callosal fibres form ectopic bundles of axons in the septum (B2 and D2) and the IG (F2) on either side of the midline (white arrowheads). Bar = 435 µm in C1, D1, E1, F1, G1, H1; 220 µm in A1, B1, C2, D2, E2, F2; 110 µm in G2, H2; 60 µm in A2, B2.

As early as E14.5, glutamatergic guidepost neurons labelled for reelin, calretinin and TBR1 failed to form a well organized band of neurons at the CSB of *Rfx3−/−* mice and instead accumulated ectopically on both sides of the midline (Figure 3A–3D; open arrowheads; Figure 4A–4D and not shown). In addition, Reelin+ Cajal Retzius and calretinin+ neurons lost dramatically their tangential distribution in the MZ layer and are more broadly distributed in the cortico-septal region (Figure 3A–3D and Figure 4A–4D). In addition, they lost their fusiform/bipolar shape. For both neuronal populations, given the density of the cells, the number of neurons in the corticoseptal region and the MZ was difficult to quantify. Similarly, from E14.5 to E16.5 glutamatergic neurons labelled for calbindin were mislocalised in the cortical MZ and IG of *Rfx3−/−* brains (Figure 3E–3F and Figure 4E and 4F). Moreover, some calbindin+ neurons were found to accumulate within the CC white matter (Figure 4E and 4F). The midline neuronal defects were accompanied, at E16.5, by pathfinding errors of pioneer callosal axons that failed to cross the midline and formed ectopic bundles (Figure 4A–4F, white arrowheads).

By contrast, GAD67-expressing GABAergic neurons were properly positioned through the lateral part of the CC at E16.5 in *Rfx3* mutant (Figure S5A and S5B). Finally, immunohistochemistry with several markers for astrocytes (nestin, GLAST and GFAP), indicated that the position and organization of the guidepost glial cell populations of the GW and of the midline glial zipper was indistinguishable in WT and *Rfx3−/−* mice, suggesting that their development is not sensitive to the loss of *Rfx3* (Figure 4G–4H and Figure S5).

Altogether, these experiments indicate that RFX3 is necessary for the proper positioning of multiple corticoseptal neuronal populations but not of glial cell populations at the midline early in development.

### Abnormal development of the CC midline in *Rfx3* mutant mice is responsible for callosal axon pathfinding defects

**Figure 5:**
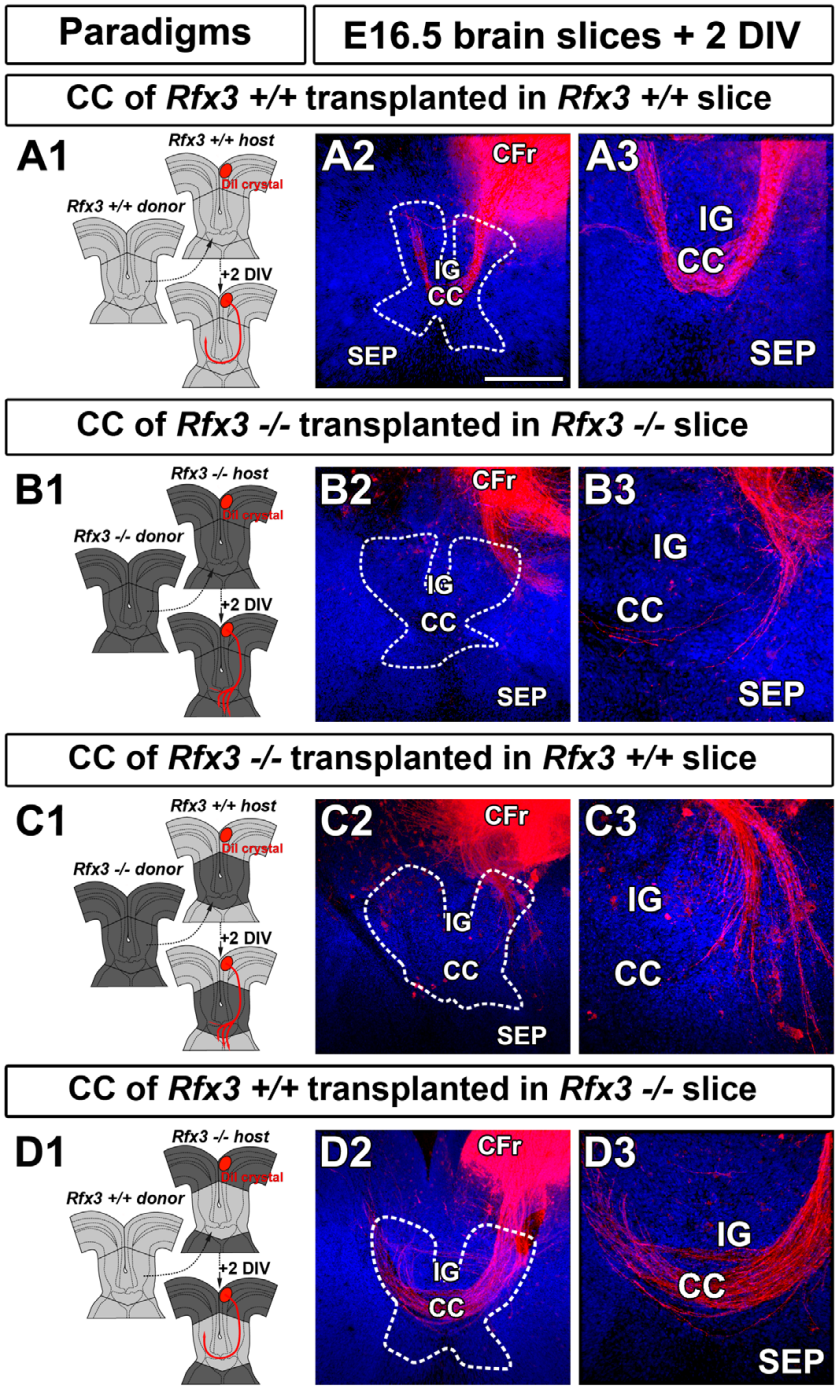
Midline integrity is necessary for pathfinding by callosal axons. (A1) Experimental paradigm used to confirm the growth of E16.5 *Rfx3+/+* control callosal axons in midline structure transplants from *Rfx3+/+* control mice. (A2–A3) DiI labeling showing that WT callosal axons grow normally and cross the midline when they are confronted to a WT environment. (B1) Experimental paradigm used to confirm the growth defects of E16.5 *Rfx3−/−* callosal axons in midline transplants from *Rfx3−/−* mice. (B2–B3) DiI labeling showing that *Rfx3−/−* callosal axons are misrouted and do not cross the midline of *Rfx3* mutants. (C1) Experimental paradigm used to study the growth defects of E16.5 control *Rfx3+/+* callosal axons in transplants of midline structures from *Rfx3−/−* mice. (C2–C3) DiI labeling showing that WT callosal axons are misrouted and do not cross the midline of *Rfx3* mutants. (D1) Experimental paradigm used to test whether the midline integrity is necessary and sufficient to direct the growth of callosal axons. To this end, control *Rfx3+/+* midline regions are transplanted in *Rfx3−/−* slices. (D2–D3) DiI labeling showing the complete restoration of *Rfx3−/−* callosal axon pathfinding. Dashed lines outline the CC transplant localizations. Brain slices in A2–A3, B2–B3, C2–C3 and D2–D3 were counterstained with Hoechst. Bar = 435 µm in A2, B2, C2, D2 and 220 µm in A3, B3, C3 and D3.

To verify if CC guidance defaults were caused by altered development in the midline region, we performed transplantation experiments as previously described [11]. Midline structures comprising the CC were transplanted into telencephalic slices at E16.5, using different combinations of wild type and *Rfx3−/−* embryos (Figure 5). When midline explants from *Rfx3+/+* mice were transplanted into *Rfx3+/+* slices, DiI-labelled callosal axons crossed the midline (Figure 5A; n = 7 slices with crossing axons out of 10), thereby reproducing the *in vivo* behavior of callosal axons. By contrast, with *Rfx3−/−* midline explants transplanted in *Rfx3−/−* slices, DiI-labelled callosal axons failed to cross the midline (Figure 5B; n = 0 slices with crossing axons out of 3). Similarly, transplantation of midline from *Rfx3−/−* mice into *Rfx3+/+* slices leads to impaired midline crossing of axons (Figure 5C; n = 2 slices with crossing axons out of 7). We then tested whether the transplantation of *Rfx3+/+* midline into *Rfx3−/−* mutant slices could restore correct pathfinding of DiI-labelled *Rfx3−/−* callosal axons. Remarkably, *Rfx3+/+* midline structure restored normal axonal guidance of the majority of *Rfx3−/−* callosal axons (Figure 5D; n = 5 slices with crossing axons out of 7).

Therefore, the misrouting of callosal axons in *Rfx3* mutant embryos is due to defects in corticoseptal midline associated structures.

### *Rfx3* inactivation in midline guidepost cells is not responsible for CC developmental defects

**Figure 6:**
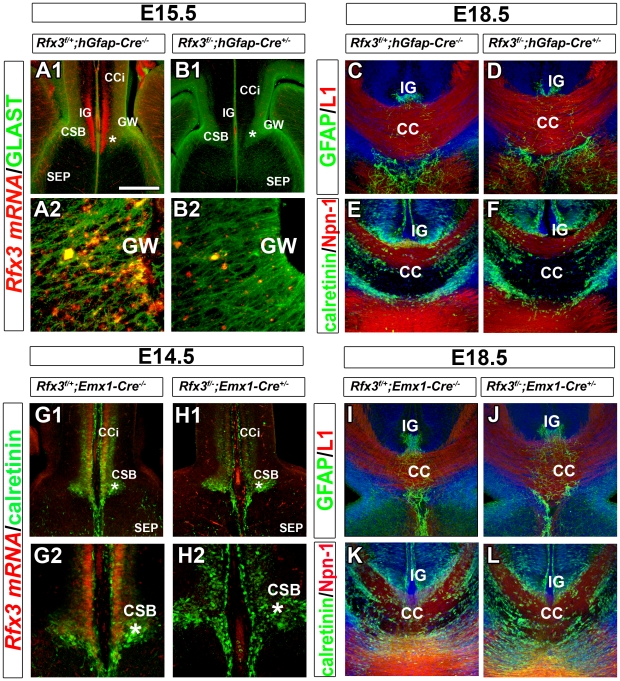
Mice carrying a conditional deletion of *Rfx3* in guidepost cells have a normal corpus callosum. (A–B) *Rfx3* mRNA (red) and GLAST protein (green) expression on coronal brain sections of control *Rfx3* ^f*/+*^ *;hGfap-Cre^−/−^* (A1–A2) and *Rfx3* ^f/−^ *;hGfap-cre^+/−^* (B1–B2) embryos at E15.5. A2 and B2 are higher magnifications of the glial wedge (GW) seen in A1 and B1, respectively. (A1–A2) In control *Rfx3* ^f*/+*^ *;hGfap-Cre^−/−^* mice, *Rfx3* is strongly expressed through the cortex, the induseum griseum (IG) and the CSB, as well as, the GW. (B1–B2) No more *Rfx3* mRNA is detected in guidepost glia and neurons of *Rfx3* ^f*/−*^ *;hGfap-Cre^+/−^* CC. (C–F) Immunohistochemistry for GFAP and L1 receptor (C and D) and for calretinin and Npn-1 receptor (E and F) in coronal CC sections from E18.5 control *Rfx3* ^f*/+*^ *;hGfap-Cre^−/−^* (C and E) and *Rfx3* ^f*/−*^ *;hGfap-Cre^+/−^* (D and F) mice. In mice where *Rfx3* is inactivated after E14.5 in midline neurons and glia, the CC and callosal axons develop normally. (G–H) *Rfx3* mRNA (red) and calretinin protein (green) expression on coronal brain sections of control *Rfx3* ^f*/+*^ *;Emx1-Cre^−/−^* (G1-G2) and *Rfx3* ^f/−^ *;Emx1-cre^+/−^* (H1–H2) embryos at E14.5. G2 and H2 are higher magnifications of the corticoseptal boundary (CSB, *) seen in G1 and H1, respectively. (G1–G2) In control *Rfx3* ^f*/+*^ *;Emx1-Cre^−/−^* mice, *Rfx3* is strongly expressed through the calretinin+ glutamatergic neurons of the cortex and of the CSB. (H1–H2) In *Rfx3* ^f*/−*^ *;Emx1-Cre^+/−^* brains, no more *Rfx3* mRNA is detected in midline glutamatergic guidepost neurons of the CSB. (I–L) Immunohistochemistry for GFAP and L1 (I and J) and for calretinin and Npn-1 (K and L) in coronal CC sections from E18.5 control *Rfx3* ^f*/+*^ *;Emx1-Cre^−/−^* (I and K) and *Rfx3* ^f*/−*^ *;Emx1-Cre^+/−^* (J and L) mice. *Rfx3* inactivation after E12.5 in guidepost glutamatergic neurons of the CSB does not affect callosal axon navigation. Bar = 435 µm in A1, B1, G1, H1; 220 µm in C, D, E, F, I, J, K, L; 110 µm in G2, H2 and 40 µm in A2, B2.

To study if RFX3 expression in guidepost glia or neurons is required for callosal axon growth, we investigated whether the targeted inactivation of *Rfx3* in both cell type progenitors might result in cell differentiation defects that could have an impact on axonal guidance. We thus inactivated *Rfx3* in GFAP-positive radial glia precursors by mating *Rfx3^f/f^* and *hGfap-Cre^+/−^* mice [29]. According to *Zhuo et al., 2001*, these mice start to express Cre recombinase in the forebrain at E13.5 [29]. We observed that while *Rfx3* was already inactivated at E15.5 in CC guidepost glia and neurons of *Rfx3* ^f/−^; *hGfap-Cre^+/−^* mice (Figure 6A and 6B), the CC still formed normally (Figure 6C–6F; n = 9/9). This result shows that the loss of *Rfx3* in midline neuronal and glial guidepost cells as early as E15.5 is not responsible for CC agenesis.

However, we cannot exclude that RFX3 is needed in the glutamatergic guidepost neurons that invade the CSB region from E12.5 to E14.5. To test this possibility, we induced recombination of *Rfx3* floxed allele specifically in guidepost neurons, as early as E12.5, by mating *Rfx3^f/f^* and *Emx1-Cre^+/−^* mice [30]. Inactivation of *Rfx3* in Emx1+ precursors of *Rfx3* ^f*/−*^ *; Emx1-Cre^+/−^* mice led to loss of *Rfx3* in all the CSB anlage at E12.5 and in midline postmitotic glutamatergic neurons at E14.5 (Figure 6G and 6H). While *Rfx3* was already inactivated at E12.5 in the CSB region of *Rfx3* ^f*/−*^ *;Emx1-Cre^+/−^*, we did not observe any callosal pathfinding defects (Figure 6I–6L; n = 6/6). These results also sustain the conclusion that *Rfx3* is not required in CC neurons. Finally, *Rfx3* was inactivated in GABAergic neurons originating from the ventral telencephalon by using the *Nkx2.1-Cre^+/−^* mice [31]. In accordance with the absence of *Rfx3* expression in CC guidepost GABAergic neurons, *Rfx3* ^f*/−*^ *;Nkx2.1-Cre^+/−^* mice did not present any CC defects (not shown).

Taken together, these results demonstrate that callosal pathfinding defects observed in *Rfx3−/−* mice are not due to a cell autonomous function of RFX3 in CC guidepost cells suggesting a requirement for RFX3 for proper CC development, at early embryonic stages, during midline specification.

### Midline specification is affected in *Rfx3−/−* forebrains

To understand how RFX3 governs CC midline structure formation before E12.5, we looked at early patterning defects that could affect *Rfx3−/−* brains. Telencephalic patterning relies on the interaction of well-described dorsal, rostral and ventral signalling centres in the forebrain that produce secreted signalling molecules [32]. We first analysed the expression of genes characteristic for the dorsal signalling centres in *Rfx3−/−* embryos by *in situ* hybridization. Genetic evidences show that *Bone morphogenic 4* (*Bmp4*) is essential for roof plate formation in the mouse forebrain [33]. BMP4 is expressed in the telencephalic midline at E10.5 and in the entire forebrain midline at E12.5. Moreover, several WNT proteins are expressed in the cortical hem [34] which is crucial for dorsal midline development. However, our analyses did not reveal any differences in *Bmp4* and *Wnt2b* expression between E12.5 WT and *Rfx3−/−* embryos (n = 2/2, Figure S6A–S6D). Thus no major defects could be observed in dorsal midline markers in *Rfx3−/−* brains.

**Figure 7:**
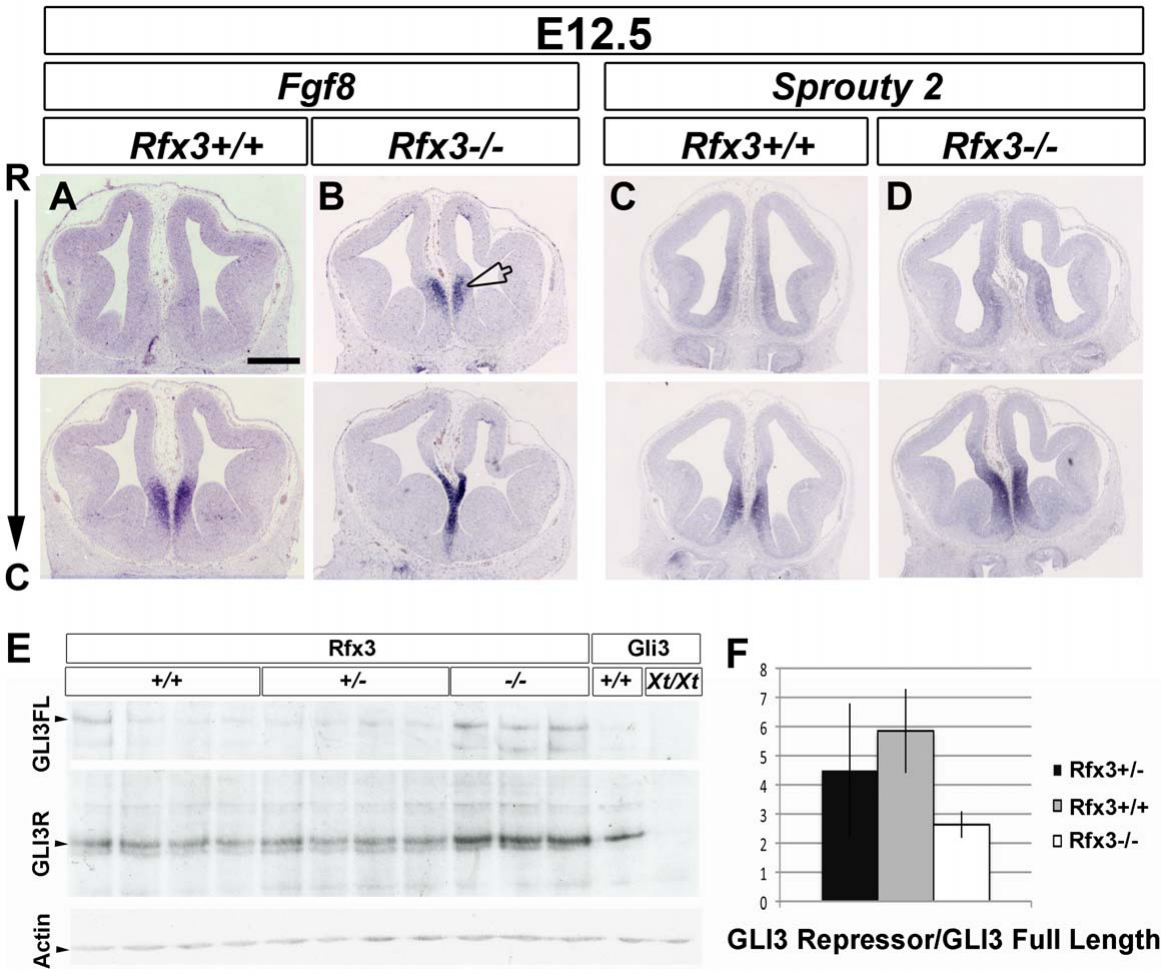
Disturbed expression of Fgf8 and of the ratio of GLI3 repressor/GLI3 activator forms in *Rfx3−/−* CSB. In situ hybridization for *Fgf8* (A and B), *Sprouty2* (C and D) mRNAs on coronal sections from E12.5 WT (A and C) and *Rfx3−/−* (B and D) mice at the CSB. *Fgf8* expression domains is expanded into the rostromedial pallium in *Rfx3−/−* embryos. Interestingly, the frontier region between the septum and the cortex is reduced in the mutant compare to WT (arrowhead). In addition, *Sprouty2* expression is slightly increased in the *Rfx3* mutant. Bar = 500 µm in all figures. (E) Western blot analysis of E13.5 individual forebrains from *Rfx3^+/+^*, *Rfx3^+/−^* and *Rfx3^−/−^* embryos from a same litter. As control, extracts from bodies of *Gli3^+/+^* or *Gli3^Xt/Xt^* embryos were included. No GLI3 protein is produced in *Gli3^Xt^* mutants allowing the identification of GLI3 specific bands. (F) Quantification of the Western blot shows that the ratio of GLI3 repressor form to the full-length form is reduced in *Rfx3* deficient mice compared to heterozygotes and WT mice.

The rostral signaling center is specified at E8.5 as the anterior neural ridge (ANR) at the anterior border between the ectoderm and neuroectoderm and will give rise to the commissural plate at later stages. Both the ANR and the commissural plate express FGF8 that has been shown to be important to induce ventral and rostrodorsal cell fates [35]. We determined the telencephalic rostral expression profile of *Fgf8* in wild type and *Rfx3−/−* embryos. As observed in Figure 7, at E12.5, *Fgf8* expression was restricted to the commissural plate of wild type embryos. Remarkably, we observed an extension of *Fgf8* expression into the rostromedial pallium in *Rfx3−/−* embryos on both coronal and sagittal sections (n = 6/6, Figure 7A, 7B and Figure S7A, S7B). These data suggest that RFX3 is necessary to restrict *Fgf8* expression to the commissural plate. FGF8 has been shown to induce *Sprouty2* gene expression which in turns negatively regulates FGF8 signalling [36], [37] and we observed a small expansion of *Sprouty2* in the rostromedial pallium consistent with an increase in FGF signalling in the midline (n = 7/7, Figure 7C, 7D and Figure S7C, S7D).

Several key transcription factors have been associated with the specification of the commissural plate in mouse, including: SIX3, nuclear factor I/A (NFIa) and EMX1 [38]. We precisely analyzed the expression of these markers but did not observe extensive variations in the expression of *Emx1* (n = 3/3), *Six3* (n = 3/3), and *Nfia* (n = 4/4) between WT and *Rfx3* mutant mice (Figure S6E–S6H and not shown) suggesting that ectopic FGF8 in *Rfx3−/−* rostral telencephalon does not induce dramatic changes in the expression of these key transcription factors.

It has been shown that proper Sonic Hedgehog (SHH) signalling is required to maintain FGF8 signaling at the rostral midline. In addition, defects in ciliary proteins lead to defective SHH signaling in many tissues and organs (for review see [39]) and also in the telencephalon [40], [41]. In comparison with *Rfx3+/+* embryos, we did not observe any differences in *Shh* expression in the ventral telencephalon of *Rfx3−/−* mutants (n = 3/3, Figure S6I and S6J). However, we observed that SHH signalling is likely to be affected since the *Shh* target genes *Patched1* (*Ptc1*) (n = 3/4) and *Gli1* (n = 2/3), were both slightly down-regulated in the *Rfx3−/−* ventral telencephalon (Figure S6K–S6N, arrows). Taken together, these findings suggest that the up-regulation of *Fgf8* expression does not coincide with an up-regulation of SHH signalling in the ventral telencephalon.

Interestingly, it has been shown that *Gli3−/−* embryos present an abnormal development of the prosencephalic midline with a similar ectopic expansion of *Fgf8* expression into the dorsal midline [42]–[44]. Since GLI3 processing has been shown to require cilia [45] and that RFX3 regulates ciliogenesis in several mouse cell types, we hypothesized that the FGF8 expression defects could result from abnormal function of GLI3 in *Rfx3−/−* embryos. *Gli3* mRNA expression did not appear to be affected in *Rfx3−/−* telencephalon, as observed by *in situ* hybridization on coronal sections (Figure S6O and S6P). Thus, RFX3 does not seem to act on *Gli3* transcription. *Gli3* produces two antagonistic protein isoforms: the full-length activator form (GLI3A) and the proteolytic cleaved repressor form (GLI3R) [46]–[48] with the ratio between GLI3A and GLI3R being an important determinant of patterning for various tissues. We thus investigated GLI3 proteolytic processing by western blot in wild type or *Rfx3* deficient brains and found that the GLI3R/GLI3A ratio is reduced in mutant brains (Figure 7E and 7F).

These results show that RFX3 is required for the proper specification of the CSB at early stages of embryonic development and that this RFX3 function is likely to be mediated by altered GLI3 processing.

### Ectopic FGF8 signalling at the CSB leads to altered organisation of CC guidepost neurons

**Figure 8:**
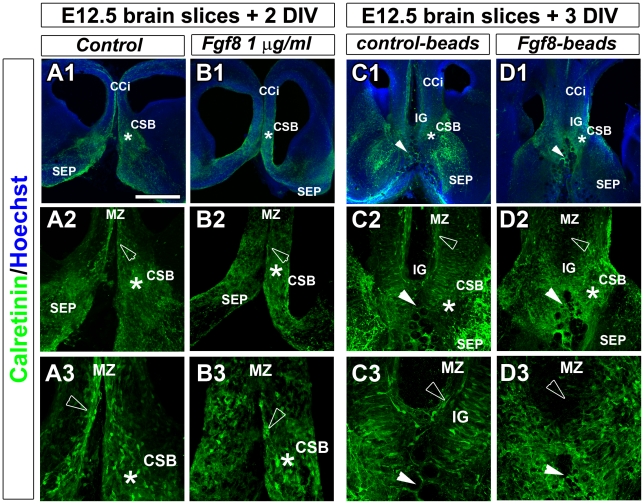
Fgf8 ectopic expression causes severe guidepost neurons mislocalization at the midline. Immunohistochemical staining for calretinin in control (A1–A3, C1–C3) and FGF8-treated (B1–B3, D1–D3) coronal CC organotypic sections. (A2–A3, B2–B3, C2–C3, D1–D3) are higher power views of the CSB region (*) seen in (A1, B1, C1, D1). In control conditions, after BSA bath application (A1–A3) or after implanting BSA-coated beads in the rostromedial pallium (white arrowheads) (C1–C3), neurons labelled with calretinin are properly positioned in cortical layers and at the CSB (*) as it is observed *in vivo*. By contrast, FGF8 bath application (B1–B3) or FGF8-coated beads implantation in the rostromedial pallium (white arrowheads) (D1–D3) results in the complete disorganization of calretinin-positive neurons that are dispersed in the entire rostromedial pallium. They failed to form a proper structure at the CSB, in the IG and to organize in layers within the cortex. They disappear at the MZ or form aggregates (arrowheads). Bar = 435 µm in A1, B1, C1, D1; 220 µm in A2, B2, C2, D2; 110 µm in A3, B3, C3, D3.

To study whether ectopic FGF8 signalling could be responsible for the disorganisation of guidepost neurons at the CSB, we performed ex-vivo cultures of brain slices at E12.5 in the presence or absence of ectopic FGF8 (Figure 8). Ectopic FGF8 sources were provided by bath application (Figure 8B) or by implanting FGF8-coated beads into the rostromedial pallium (Figure 8D) where the extension of *Fgf8* expression was observed in *Rfx3−/−* embryos. We followed the distribution of guidepost neurons 2–3 days later by immunostaining for Calretinin. Remarkably, under both conditions, we observed drastic consequences of ectopic FGF8 on guidepost distribution at the midline in treated explants (n = 10/10 after FGF8 bath application and n = 6/6 with FGF8-coated beads) compared to control (n = 11/11 without FGF8 bath application and n = 5/5 with control-coated beads). The observed phenotypes were similar to what is observed in *Rfx3−/−* brains. The tangential distribution of Calretinin+ neurons through the cortical MZ was severely reduced and neurons were broadly distributed at the CSB and in cortical layers. No MZ could be clearly distinguished. We also noticed a marked thinning of the commissural plate as it was observed in several *Rfx3* mutant mice compared to WT mice (Figure 7B and white arrowheads in Figure S6). These results support the hypothesis that FGF8 controls guidepost neuronal distribution at the CSB. Hence, in *Rfx3−/−* brains, the observed increase in *Fgf8* expression can be indirectly responsible at early stages for disturbed guidepost neuron distribution by acting on CSB patterning, but also directly responsible at later stages for the positioning of guidepost neurons at the CSB. Therefore, loss of CC in *Rfx3−/−* mice likely results from the perturbed processing of GLI3 which controls *Fgf8* expression early during development.

## Discussion

*Rfx3−/−* mouse mutant provides a valuable tool for dissecting the relative contributions of early brain patterning mechanisms in the organization of the CSB and in CC formation. We show that *Rfx3−/−* mice have mild and focused FGF8 over expression that is restricted to the CSB and that severe CC defects coincide with this early patterning anomaly. In turn, this patterning defect is responsible for dramatic changes in the distribution of guidepost neurons but not of glial cells at the corticoseptal midline in *Rfx3−/−* mice. In addition, our transplantation experiments support the conclusion that proper guidepost neuronal network organization mediated by FGF8 signalling at the midline is essential for proper CC formation.

### RFX3 and guidepost cells of the corticoseptal boundary

RFX3 is strongly expressed in corticoseptal guidepost cells from E14.5 to E16.5. However, we did not observe any consequences of *Rfx3* loss of function on CC formation by *Emx1-Cre* or *hGFAP-Cre* induced *Rfx3* inactivation in these cells, suggesting that RFX3 function in guidepost cells is not required for commissural axon formation or that RFX3 function in these cells is masked by a redundant function of other RFX transcription factors. Indeed, 7 RFX proteins are present in the mouse genome and at least one, RFX4, has already been shown to play major functions in brain patterning and to be widely expressed in the telencephalon [49], [50]. However, no precise description of RFX4 expression in guidepost neurons or glial cells have been described and CC callosum defects have not been precisely investigated in RFX4 mutants. Hence, the function of RFX proteins in guidepost cells still remains to be deciphered. Nevertheless, our work shows that RFX3 plays a crucial function in early patterning of the CSB.

### Increased FGF8 signalling disturbs distribution of guidepost neurons required for CC formation

We show that RFX3 is required early to restrict FGF8 expression in the telencephalic rostral midline. Our results suggest that a small increase in *Fgf8* signalling is sufficient to disorganize the CSB and hence guidepost neuron distribution at early stages of embryonic development. This is supported by *in-vivo* observations on *Rfx3* mutant brains. On the other hand, our *ex-vivo* explant experiments support the hypothesis that FGF8 also has a direct action on guidepost neuron distribution at later stages. Altogether, these observations show that FGF8 plays a critical function for the distribution of guidepost neurons at the CSB. Previous data in the literature indicated that reducing FGF8 signalling in the telencephalon leads to severe brain phenotypes, and in particular to holoprosencephaly [35], [51], [52] and *Fgf8* hypomorphic mutants show corpus callosum defects [51]. Moreover *Fgf8* inactivation by *Emx1*-*Cre* induced recombination has been shown to induce CC defects at E18.5, suggesting a key role for FGF8 in CC formation [38]. In all these studies, while FGF8 signalling defects were associated with severe alterations of the dorsal signalling centres and possible defects at the CSB, the distribution of guidepost neurons was not examined. In *Rfx3−/−* mice, unbalanced FGF8 expression in the rostral telencephalon is not associated with alterations of the dorsal signalling centre but is nevertheless sufficient to disturb the distribution of guidepost neurons, leading to the conclusion that FGF8 signalling is primarily responsible for guidepost neuron organization at the CSB and hence CC formation.

Our work also indicates that RFX3 acts on FGF8 signalling by regulating GLI3 activity in the telencephalon. This in agreement with previous works showing that GLI3 acts upstream of FGF8 signalling in the telencephalon. Indeed, in *Shh* mutants *Fgf8* expression is lost, whereas in *Gli3* mutants *Fgf8* expression is expanded [42]–[44]. Moreover, in *Shh*; *Gli3* double mutants, *Fgf8* expression is also expanded suggesting that this expansion occurs independently of *Shh* in a *Gli3* mutant background [44]. Last, loss of *Gli3* rescues ventral patterning defects in *Shh* mutants but not in *Fgfr1*;*Fgfr2* mutants, placing FGF signalling downstream of *Gli3* [53]. This also explains why the upregulation of *Fgf8* expression likely occurs in *Rfx3−/−* brains despite an observed downregulation of *Shh* signalling. Our observations and these data are consistent with the conclusion that *Rfx3* acts on GLI3 activity and consequently on FGF8 signalling at the rostral telencephalic midline. Interestingly, our work brings a mechanistic interpretation to the observation that *Gli3* mutations have been found in human patients suffering from Acrocallosal syndrome [54]. In addition, hypomorphic *Gli3^Pdn^* mutant mice show CC defects [55] with a similar but more severe increase in FGF8 expression in the rostral midline [42]. Consistent with our observations, *Gli3^Pdn^* mutants also show strong alterations of guidepost neuronal organization (D. Magnani and T. Theil, unpublished).

### Ciliary proteins and corpus callosum defects

In *Rfx3−/−* brains, we observed a reduced processing of GLI3 in its repressor form and a reduction in SHH signalling in agreement with the already described function of RFX3 in ciliogenesis in various cell types. Indeed, the function of cilia in regulating SHH signalling and GLI3 processing has been well documented in various cell types and organs (for review see [39]). Interestingly, RFX proteins have been shown to control consistently the regulation of several IFT components, dynein retrograde motors and many basal body associated proteins such as MKS1 from *C. elegans* to mammals [17], [56], [57]. Many of these RFX target genes appear to be associated with overall reduced SHH signalling and reduced GLI3 processing when mutated in mouse. These observations suggest that RFX3 indirectly modulates GLI3 activity and SHH signaling in the anterior telencephalon by regulating the levels of several proteins involved in cilia associated transport and biogenesis.

In humans, several syndromes resulting from mutations in genes encoding ciliary proteins are associated with corpus callosum malformation of various severity [15]. Recently, mutations in the *Kif7* gene involved in ciliogenesis and GLI3 processing have been found in human patients suffering from acrocallosal syndrome, characterized by Corpus Callosum and digit malformations [58]. Our work provides a first insight into the cellular mechanisms that are responsible for Corpus Callosum defects following GLI3 processing alterations. Our work demonstrates that small alterations in GLI3 processing is correlated with altered patterning of the CSB and aberrant distribution of guidepost neurons in this region and that this is sufficient to induce midline crossing defects of callosal axons. In mouse, only a few ciliary mutants with altered patterning of the telencephalon have been described. The cobblestone hypomorphic mutation of *Ift88*, and the *ftm*, and *alien* mutants all show severe morphological defects of the brain associated with dorsal ventral patterning defects of the telencephalon [40], [41], [59]. All three mutants are associated with an alteration in GLI3 processing, but with a more severe shift in the balance of GLI3 activator and repressor forms than in *Rfx3* mutants. However, the CC was not precisely investigated in these mutants, probably because embryos die too early to allow for an analysis of CC development. Another interesting mutant is a *Wnt1-Cre* induced *Kif3A*-deleted mouse that shows craniofacial anomalies due to neural crest migration defects and is associated with agenesis of the CC [60]. Neural crest migration is required for the proper patterning of the telencephalon, in particular by acting on FGF8 rostral patterning centre [61], [62]. The patterning of the telencephalon has not been described in *Wnt1-cre; Kif3A^flox/flox^* mice but it is tempting to hypothesize that dysregulation of FGF8 signalling could be sufficient to mis-pattern the commissural plate in this mutant. Hence, the *Rfx3−/−* mutant represents the first mouse model establishing a link between proper GLI3 processing and the distribution of guidepost neurons at the CSB for CC formation.

In conclusion, the analysis of *Rfx3−/−* mice provides strong evidence for the important contribution of corticoseptal neuronal populations in CC formation and for the critical function of *Fgf8* signalling in CSB patterning at early stages of CC formation. It provides new understanding of the cellular origins of CC defects in human ciliopathies.

## Materials and Methods

### Animals

All animal research has been conducted according to relevant national and international guidelines. *Rfx3*-deficient and floxed mice were generated and genotyped as previously described [18]. *GAD67-GFP* knock-in mice, *hGfap-Cre^+/−^*, *Ngn2-creER^TM+/−^*, *Emx1-cre^+/−^*, *Nkx2.1-cre^+/−^* mice used in this work have been previously described [27], [29]–[31].

### Histology

Brains were fixed in 4% PFA/PBS at 4°C until experimentation and then dehydrated in graded series of ethanol (25–100%). Brains were transferred into absolute butanol and substituted for paraffin in graded series of butanol/paraffin solutions. Sections of 10 µm were deparaffinized in Methylcyclohexan, rehydrated and stained with Hematoxylin following standard procedures before mounting in Eukitt.

### Immunocytochemistry

Brain embryos were dissected and fixed overnight at 4°C in 4% paraformaldehyde (PFA) (Sigma P6148) in 1×PBS (Invitrogen). Brains were cryoprotected in 30% sucrose and cut in coronal 50 µm-thick frozen sections for staining. Mouse monoclonal antibodies were: Nestin (1/600) (BD bioscience). Rat monoclonal antibodies were: L1 (1/200) (Chemicon, Temecula, CA) and CTIP2 (1/500) (Abcam, Cambridge, UK). Rabbit polyclonal antibodies were: calbindin (1/2500) and calretinin (1/2000) (Swant, Bellinzona, Switzerland); CUX1 (1/200) (Santacruz, Heidelberg, Germany); EMX1 (1/250) (gift form A. Trembleau); GFAP (1/500) (DAKO, Carpinteria, CA); GFP (Molecular Probes, Eugene, OR); SATB2 (1/500) (gift from V. Tarabykin); RFX3 (1/100) [63], TBR1 (1/500) (Abcam, Cambridge, UK). Goat polyclonal antibody was Npn-1 (1/50) (R&D System, Minneapolis, Mn). GLAST guinea-pig polyclonal antibody was (1/2000) (Millipore, Billerica, MA). GFP chicken polyclonal antibody was (1/500) (AVES, Oregon, USA).

Fluorescence immunostaining: The primary antibodies were detected with secondary antibodies coupled with Cy or Alexa (Jackson ImmunoResearch and Molecular Probes; respectively). For RFX3 detection, we used an amplification system with secondary anti-rabbit IgG coupled to biotin (1/250) (Jackson Laboratory) and subsequent revelation with Streptavidin Fluoprobes 547 (Interchim) (1/400). Sections were counterstained with Hoechst 33258 (Molecular Probes), mounted on glass slides and covered in Mowiol 4–88 (Calbiochem, Bad Soden, Germany).

### *In situ* hybridization on cryosections

Embryos were fixed overnight at 4°C in 4% PFA in PBS. Embryos were transferred in PBS/30%sucrose, cryoprotected in PBS/15% Sucrose (Sigma S0389)/7.5% Gelatin (Merck 4078) and frozen at −50°C. Cryosections of 20 µm were collected, thaw-mounted on polylysin coated slides (Miom France). Hybridization was performed as previously described (Niquille et al., 2009). *Rfx3* mRNA probe was previously described [18].

### *In situ* hybridization combined with immunocytochemistry

Embryonic brains were treated as described for immunocytochemistry procedure and coronal 100 µm-thick sections were cut using a vibratome (Leica Microsystems). 100 µm free-floating vibratome sections were hybridized with digoxigenin-labeled cRNA probe as described before [64]. To combine *in situ* hybridization with immunocytochemistry, fast Red (Roche) was used as an alkaline phosphatase fluorescent substrate instead of NBT/BCIP solution. Slides were incubated in Fast Red (Roche) until the appearance of staining in dark chamber at RT. Thereafter, sections were fixed for 15 min in 4% PAF and immunostaining was performed.

### Imaging

Fluorescent-immunostained sections were imaged using confocal microscope (Zeiss LSM 510 Meta, Leica SP5 or Zeiss Qasar 710) equipped with 10×, 20×, 40×oil Plan-NEOFLUAR and 63×oil, 100×oil Plan-Apochromat objectives. Fluorophore excitation and scanning were done with an Argon laser 458, 488, 514 nm (blue excitation for GFP and Alexa488), with a HeNe laser 543 nm (green excitation for Alexa 594, CY3 and DiI), with a HeNe laser 633 nm (excitation for Alexa 647 and CY5) and a Diode laser 405 nm (for Hoechst staining). Z-stacks of 10–15 plans were acquired for each CC coronal section in a multitrack mode avoiding crosstalk.

Images processing: all 3D Z stack reconstructions and image processing were performed with Imaris 6.0 software. To create real 3D data sets we used the mode “Surpass”. The colocalization between two fluorochromes was calculated and visualized by creating a yellow channel. Figures were processed in Adobe Photoshop CS2.

### Western blot

Western blots were performed following standard procedures. Gli3 6F5 mouse monoclonal antibody was kindly provided by S. Scales [65]. Gli3XT and WT body samples were kindly provided by M. Willaredt and S. Schneider-Maunoury.

### Slice culture experiments

We have used our previously developed in vitro model of CC organotypic slices [11]. Embryos were placed in ice cold dissecting medium (MEM Gibco ref 11012-044 with 15 mM glucose and 10 mM Tris pH 7–9). Brains were removed and embedded in 3% low-melting point agarose (In vitrogen). 250 µm thick coronal sections were then cut using a vibrotome filled with cold dissecting medium and slices at the level of the CC were collected in the same medium. CC slices were cultured on Millicell membranes in tissue dishes containing 1 ml of BME/HBSS (Invitrogen) supplemented with glutamine, 5% horse serum, and Pen/Strep [54]. In our slice assay, as *in vivo*, the callosal axons from dorso-lateral neocortex develop later and their growth cones enter after E16.5 the CC region in successive streams over a period of several days. Our slice assay performed at E16.5 allowed us to study: (1) the function of CSB cells, (2) the outgrowth properties of the majority of callosal axons that are growing through the CC after E16.5 and (3) the effects of transplantations on callosal axon navigations.

The transplantation assay was performed at E16.5 as previously described [11] to analyze the growth of either WT or *Rfx3−/−* DiI-labelled callosal axons within midline structures of WT or *Rfx3−/−* slices. Small explants of E16.5 WT or *Rfx3−/−* midline structure comprising were excised using tungsten needles and transplanted into the midline of WT or *Rfx3−/−* host slices. After incubation for 48 hours, the slices were fixed and axon trajectories through the various regions were analysed by confocal analysis. For FGF8 bath application: slices of E12.5 brains were cultured as above but FGF8 (FGF-8b isoform, R&D Systems, ref 423-F8) or BSA (control) was added in the culture medium at 1 µg/ml in PBS 1×. After two days of incubation, slices were processed for immunohistochemistry as described above. For FGF8-coated beads experiments: slices of E12.5 brains were cultured as above but FGF8-coated beads or control-coated beads were implanted in the rotromedial pallium. After three days of incubation, slices were processed for immunohistochemistry as described above. To make FGF8-soaked beads, 45 µm polystyrene beads (Polysciences) were rinsed in PBS, the beads were pelleted by 5-min centrifugation at 13,000 rpm and the supernatant was removed. They were incubated in 5 mg/ml heparin for 1 hour at room temperature, rinsed and then incubated with 250 µg/ml mouse FGF8b (R&D Systems) in 0.5% bovine serum albumin (BSA) in PBS overnight at 4°C. Control beads were incubated in 0.5% BSA in PBS. Before implantation, beads were rinsed three times for 10 minutes in PBS.

### Atlas and nomenclature

The nomenclature for callosal development is based on the “Atlas of the prenatal mouse brain” [66].

## Supporting Information

Figure S1 Defects of the CC and hippocampal commissure in *Rfx3−/−* brains at E18.5. (A–C) Haematoxylin-eosin staining performed at different rostro-caudal (R→C) levels on coronal brain sections of E18.5 wild type (A1–A2) or *Rfx3−/−* (B1–B2 and C1–C2) embryos. (A1–A2) At E18.5, the hemispheres of the WT brain have fused. The CC and the hippocampal commissure (HIC) are already formed. (B and C) Around 70% of *Rfx3−/−* embryos show either a partial CC agenesis with few callosal axons crossing (B1–B2) or a complete CC agenesis with an absence of midline fusion (O) and no midline crossing (C1–C2). All callosal defects are associated with Probst Bundle (PB) formation (arrowheads). The hippocampal commissure (HIC) development is also affected in most mutants either as a reduction or a complete loss of this commissure (C2). Bar = 600 µm in all.
(TIF)

Figure S2 Numerous RFX3-positive neuronal populations are found within the embryonic cingulate cortex and at the corticoseptal boundary. (A–G) *In situ* hybridizations for *Rfx3* (in red) combined with immunohistochemical staining for reelin (A1–A2), TBR1 (B1–B2), calretinin (C1–C2 to E1–E2) or calbindin (F1–F2 and G1–G2) (in green) on coronal brain sections in WT mice at E13.5 (C1–C2), E14.5 (A1–A2 and B1–B2), E15.5 (D1–D4 and F1–F2) and E16.5 (E1–E2 and G1–G2). A2, B2, C2, D2, D4, E2, F2 and G2 are high-power views of the midline region seen in A1, B1, C1, D1, D3, E1, F1 and G1, respectively. (A1–A2) *Rfx3+* cells of the marginal zone (MZ) within the cingulate cortex (CCi) are not reelin+ Cajal Retzius cells. (A1) Reelin is expressed in the corticoseptal region (CSB, *). (B1–B2) *Rfx3+* cells residing in the CSB and in the cortex express TBR1. (B2) All neurons that contain high levels of cytosolic *Rfx3* mRNAs express the nuclear TBR1 transcription factor (arrowheads). Therefore, *Rfx3+* cells of the CSB and of the cortex are glutamatergic neurons. (C to E) Co-labeling experiments performed, from E13.5 to E16.5, show that the majority of *Rfx3*-expressing neurons in the cortical MZ (C1–C2, D3–D4 and E2), as well as, in the CSB (D1–D2) express calretinin (arrowheads). After E16.5, while the cerebral hemispheres have fused and the CC is formed, *Rfx3* expression persists in calretinin+ neurons of the cortex (E2; arrowheads) but stops in calretinin^+^ guidepost neurons (E1; arrow). (F1–F2 and G1–G2) From E15.5 to E16.5, the *Rfx3*+ neurons of the IG express the calbindin. By contrast, *Rfx3*+ neurons of the MZ do not express calbindin. Bar = 220 µm in A1, B1, C1, D1, E1, F1, G1; 110 µm in C2, D3; 60 µm in A2, G2 and 40 µm in B2, D2, D4, E2, F2.
(TIF)

Figure S3 Expression of *Rfx3* in radial glia precursors of the GW and in cortical pyramidal neurons. (A1–A2) Immunohistochemistry for calretinin and Emx1 in coronal sections from mice at E15.5. A2 is a higher magnification of the midline corticoseptal boundary (CSB, *) seen in A1. Calretinin+ neurons of the CSB are glutamatergic since they express EMX1 (arrowheads in A2). (B1–B2) *In situ* hybridizations for *Rfx3* (red) combined with immunohistochemical staining for Calbindin (green) on coronal brain sections in WT mice at E16.5. Calbindin+ neurons of the CSB are glutamatergic since they express *Emx1* (arrowheads in B2). (C1 and C2) Immunohistochemical staining for RFX3 in coronal sections from GAD67-GFP transgenic mice at E18.5 showing that RFX3 is not expressed by GABAergic interneurons. (D–H) *In situ* hybridizations for *Rfx3* (red) combined with immunohistochemical staining for Nestin (D1–D2), GFAP (E1–E2 and F1–F2), SATB2 (G1–G2) or CTIP2 (H1–H2) (green) on coronal brain sections in WT mice at E14.5 (D1–D2), E16.5 (E1–E2) and E18.5 (F1–F2 to H1–H2). D2, E2, F2, G2 and H2 are high magnifications of D1, E1, F1 G1 and H1, respectively. (D1–D2 to F1–F2) Radial glial cells of the glial wedge (GW) labelled for Nestin and GFAP express high levels of *Rfx3* (arrowheads). By contrast, astrocytes of the indusium griseum (IG) labelled for the same markers are devoided of *Rfx3*. (G1–G2 and H1–H2) At E18.5, *Rfx3* is expressed by callosal pyramidal neurons (SATB2+) and by sub-cerebral projecting neurons (CTIP2+). (G2 and H2) High magnified views of the cortex showing that glutamatergic pyramidal neurons expressing the nuclear transcription factors SATB2 or CTIP2 contain cytosolic *Rfx3* mRNAs (arrowheads). Bar = 435 µm in A1, G1, H1; 220 µm in B1, D1, E1; 110 µm in C1, C2; 60 µm in A2, B2; 40 µm in D2, E2, F1, F2, G2, H2.
(TIF)

Figure S4 *Rfx3* inactivation in cortical pyramidal neurons is not responsible for callosal axon guidance defects. (A–H) Single immunohistochemistry for SATB2 (A and B), for CUX1 (C and D), for TBR1 (E and F) or for CTIP2 (G and H) in coronal sections from E18.5 WT (A, C, E and G) and *Rfx3* ^−/−^ (B, D, F and H) mice. SATB2+, CUX1+, TBR1+ and CTIP2+ cortical layers are not affected in the *Rfx3^−/^* ^−^. (I–J) *In situ* hybridizations for *Rfx3* (in red) combined with immunohistochemical staining for SATB2 (in green) on coronal brain sections of control *Rfx3* ^f*/+*^ *;Ngn2-CreERtm^−/−^* (I1–I2) and *Rfx3* ^f/−^ *;Ngn2-CreERtm^+/−^* (J1–J2) embryos at E18.5. (I1–I2) In control *Rfx3* ^f*/+*^ *;Ngn2-CreERtm^−/−^* mice, *Rfx3* is strongly expressed through the glutamatergic neurons of the cortex and of the indusium griseum (IG). (J1–J2) In *Rfx3* ^f/−^ *;Ngn2-CreERtm^+/−^* brains, *Rfx3* hybridation signal is significantly decreased in all the cortical layers after induced recombination of *Rfx3* floxed allele in Ngn2-derived cells. *Rfx3* inactivation in the cortex does not affect the cortical distribution of SATB2+ callosal projecting neurons. (K–L) Double immunohistochemistry for calretinin and Npn-1 (K1–K2 and L1–L2) in coronal CC sections from E18.5 control *Rfx3* ^f*/+*^ *;Ngn2-CreERtm^−/−^* (K1–K2) and *Rfx3* ^f/−^ *;Ngn2-CreERtm^+/−^* (L1–L2) mice. In brain sections of mice where *Rfx3* is conditionally inactivated in Ngn2-derived cortical pyramidal neurons, callosal axons develop normally. Bar = 435 µm in I1, J1, I2, J2, K1, L1; 220 µm in K2, L2 and 110 µm in A, B, C, D, E, F, G, H.
(TIF)

Figure S5 Localization of GABAergic interneurons in *Rfx3* deficient mice. (A–B) *In situ* hybridization for *Gad67* mRNAs on coronal CC slices of E16.5 *Rfx3* deficient mice (B1–B2) in comparison with wild type (A1–A2). We observe a normal localization of *Gad67* in telencephalon, and notably in the lateral CC region, of *Rfx3* deficient mice (B1–B2) compared to wild type mice (A1–A2). (C–D) DAB staining for GLAST on coronal rostromedial slices from E16.5 WT (C) and *Rfx3*−/− (D) mice. We observe a normal localization and organization of guidepost glia of the midline zipper glia (MZG) and of the glial wedge (GW) of *Rfx3* deficient mice (D) compared to wild type mice (C). Bar = 1200 µm in A1, B1, Bar = 300 µm in C, D, and 150 µm in A2, B2.
(TIF)

Figure S6 Expression of dorsal and ventral markers in *Rfx3−/−* brains. In situ hybridization for *Shh* (A and B), *Gli1*(C and D), *ptc1*(E and F), *Gli3* (G and H), *Bmp4* (I and J) and *Wnt2b* (K and L) mRNAs on coronal sections from E12.5 WT (A, C, E, G, I, K, M and O) and *Rfx3*−/− (B, D, F, H, J, L, N and P) mice. (A to D) All dorsal midline markers are normally expressed in the *Rfx3* ^−/−^ embryos. (E to H) All commissural plate markers are normally expressed in the *Rfx3* ^−/−^ embryos. (I to P) While *Shh* and *Gli3* were properly specified, *Ptc1* receptor and *Gli1* are down-regulated (black arrows) in *Rfx3*−/−. Interestingly, the frontier region between the septum and the cortex is reduced in some mutants compare to WT (empty arrows). Bar = 500 µm in all.
(TIF)

Figure S7 Enlargement of the *Fgf8* expression domain in *Rfx3−/−* embryos. In *situ hybridization* for *Fgf8* (A and B) and *Sprouty2* (C and D) mRNAs on sagittal midline sections from E12.5 WT (A and C) and *Rfx3−/−* (B and D) embryos at the CSB. *Fgf8* and *Sprouty2* expression domains are expanded rostrally and laterally in the pallium of *Rfx3−/−* embryos (arrows). Bar = 500 µm in all figures.
(TIF)
