# Supplementary figures and images for: The Ciliogenic Transcription Factor RFX3 Regulates Early Midline Distribution of Guidepost Neurons Required for Corpus Callosum Development
Source: PLoS Genet. 2012 Mar 29;8(3):e1002606. doi: 10.1371/journal.pgen.1002606 (PMC3315471; doi:10.1371/journal.pgen.1002606)

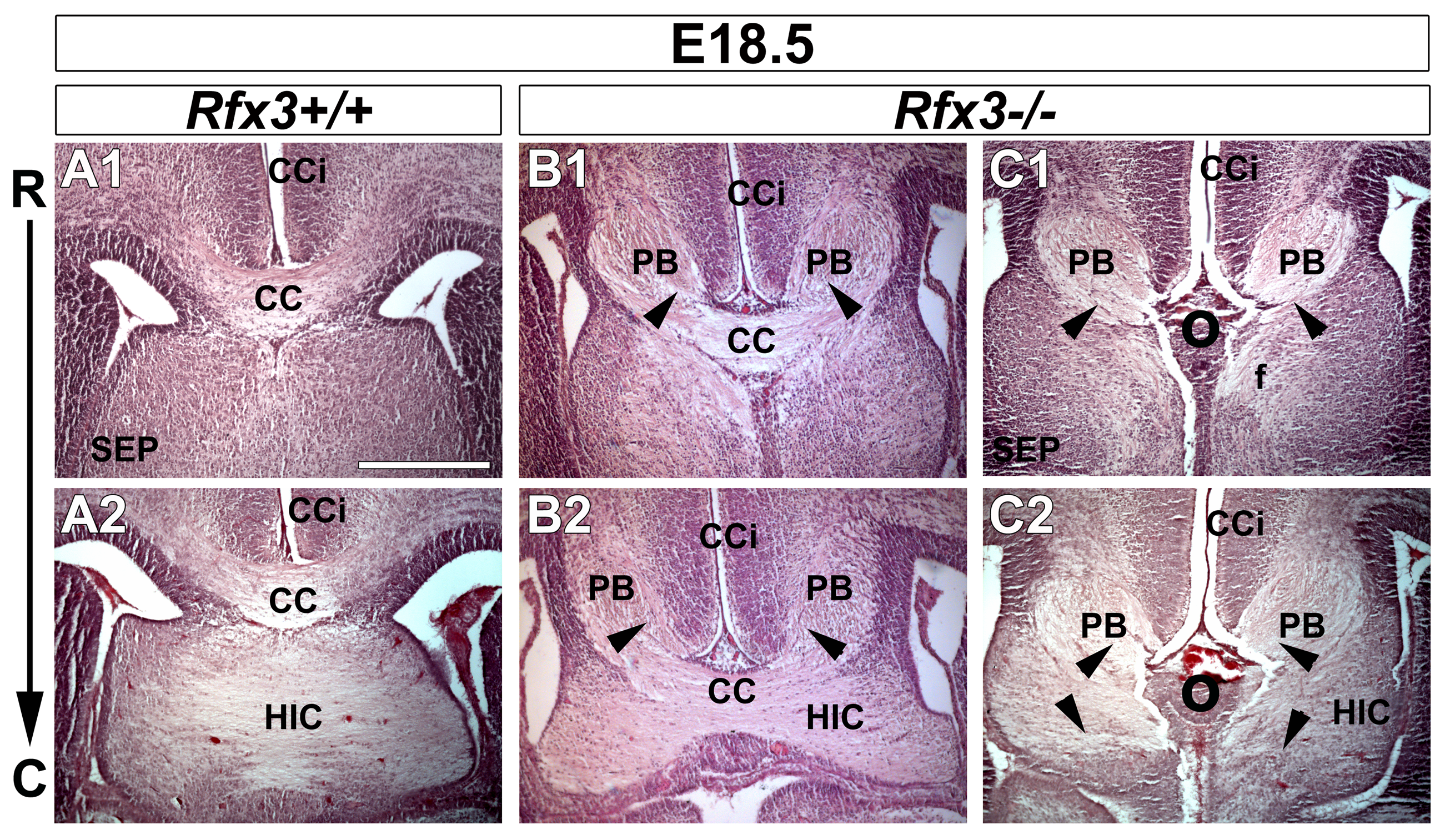

Supplement: Figure S1 — Defects of the CC and hippocampal commissure in Rfx3−/− brains at E18.5. (A–C) Haematoxylin-eosin staining performed at different rostro-caudal (R→C) levels on coronal brain sections of E18.5 wild type (A1–A2) or Rfx3−/− (B1–B2 and C1–C2) embryos. (A1–A2) At E18.5, the hemispheres of the WT brain have fused. The CC and the hippocampal commissure (HIC) are already formed. (B and C) Around 70% of Rfx3−/− embryos show either a partial CC agenesis with few callosal axons crossing (B1–B2) or a complete CC agenesis with an absence of midline fusion (O) and no midline crossing (C1–C2). All callosal defects are associated with Probst Bundle (PB) formation (arrowheads). The hippocampal commissure (HIC) development is also affected in most mutants either as a reduction or a complete loss of this commissure (C2). Bar = 600 µm in all. (TIF) [file pgen.1002606.s001.tif]

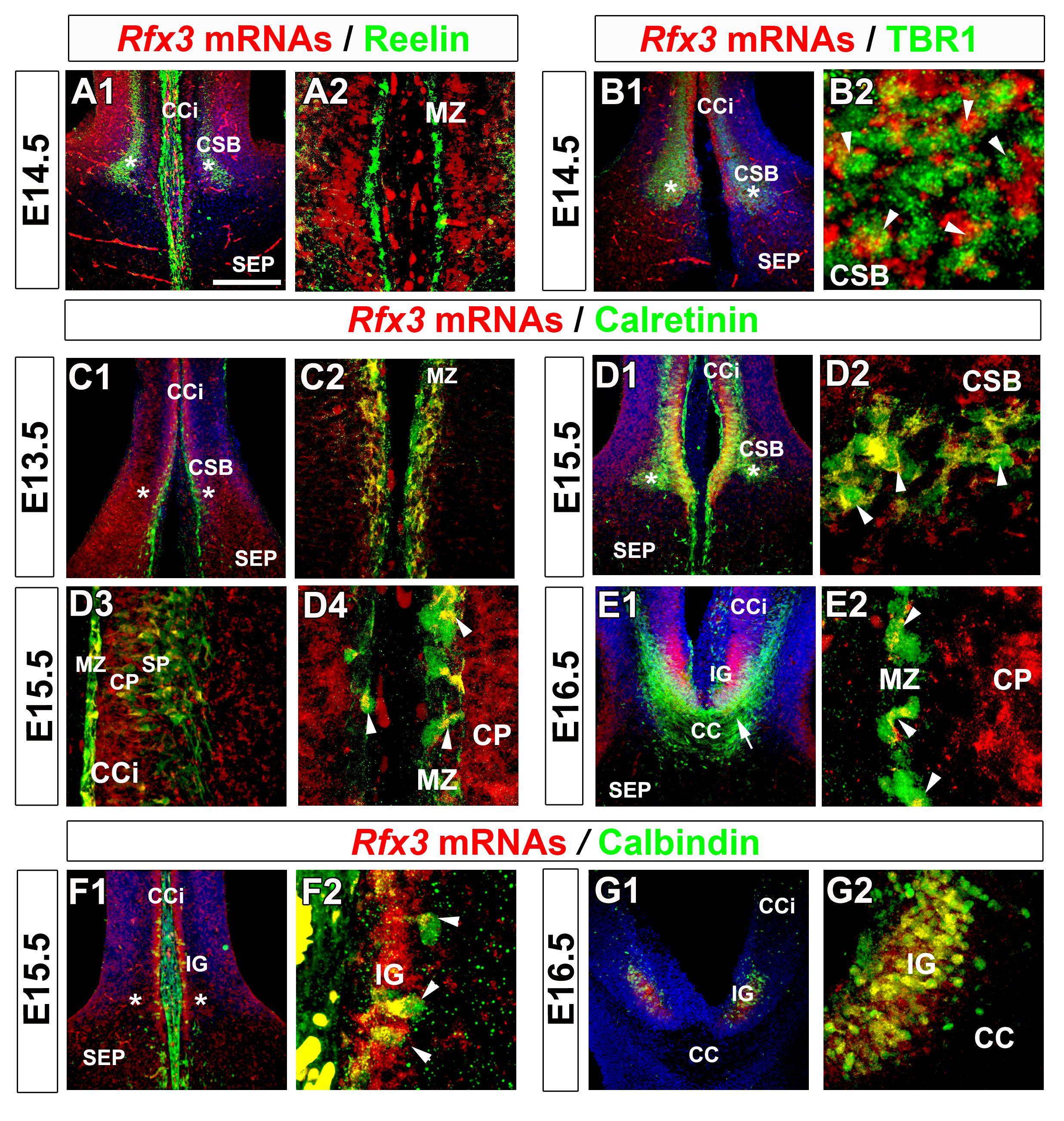

Supplement: Figure S2 — Numerous RFX3-positive neuronal populations are found within the embryonic cingulate cortex and at the corticoseptal boundary. (A–G) In situ hybridizations for Rfx3 (in red) combined with immunohistochemical staining for reelin (A1–A2), TBR1 (B1–B2), calretinin (C1–C2 to E1–E2) or calbindin (F1–F2 and G1–G2) (in green) on coronal brain sections in WT mice at E13.5 (C1–C2), E14.5 (A1–A2 and B1–B2), E15.5 (D1–D4 and F1–F2) and E16.5 (E1–E2 and G1–G2). A2, B2, C2, D2, D4, E2, F2 and G2 are high-power views of the midline region seen in A1, B1, C1, D1, D3, E1, F1 and G1, respectively. (A1–A2) Rfx3+ cells of the marginal zone (MZ) within the cingulate cortex (CCi) are not reelin+ Cajal Retzius cells. (A1) Reelin is expressed in the corticoseptal region (CSB, *). (B1–B2) Rfx3+ cells residing in the CSB and in the cortex express TBR1. (B2) All neurons that contain high levels of cytosolic Rfx3 mRNAs express the nuclear TBR1 transcription factor (arrowheads). Therefore, Rfx3+ cells of the CSB and of the cortex are glutamatergic neurons. (C to E) Co-labeling experiments performed, from E13.5 to E16.5, show that the majority of Rfx3-expressing neurons in the cortical MZ (C1–C2, D3–D4 and E2), as well as, in the CSB (D1–D2) express calretinin (arrowheads). After E16.5, while the cerebral hemispheres have fused and the CC is formed, Rfx3 expression persists in calretinin+ neurons of the cortex (E2; arrowheads) but stops in calretinin+ guidepost neurons (E1; arrow). (F1–F2 and G1–G2) From E15.5 to E16.5, the Rfx3+ neurons of the IG express the calbindin. By contrast, Rfx3+ neurons of the MZ do not express calbindin. Bar = 220 µm in A1, B1, C1, D1, E1, F1, G1; 110 µm in C2, D3; 60 µm in A2, G2 and 40 µm in B2, D2, D4, E2, F2. (TIF) [file pgen.1002606.s002.tif]

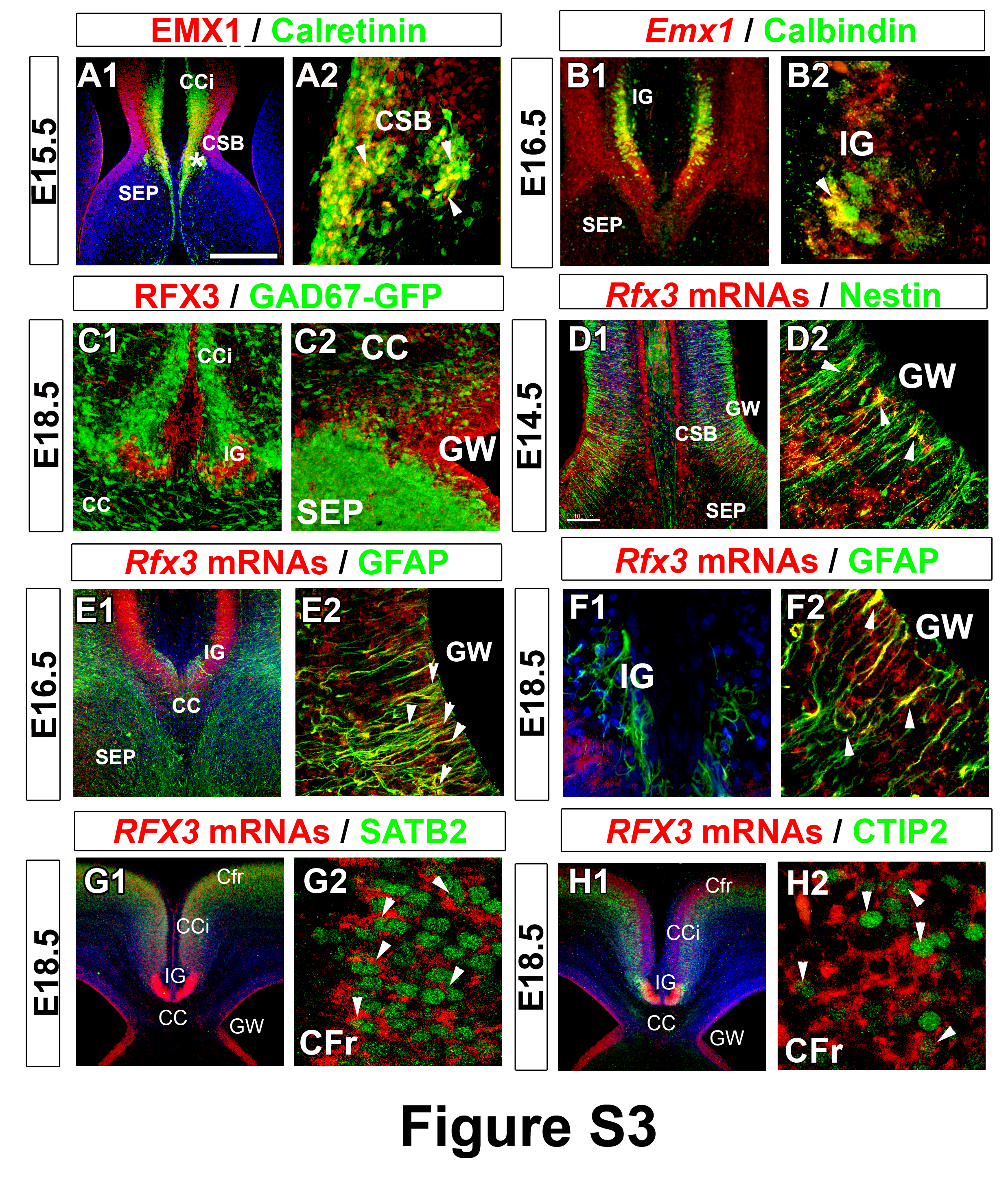

Supplement: Figure S3 — Expression of Rfx3 in radial glia precursors of the GW and in cortical pyramidal neurons. (A1–A2) Immunohistochemistry for calretinin and Emx1 in coronal sections from mice at E15.5. A2 is a higher magnification of the midline corticoseptal boundary (CSB, *) seen in A1. Calretinin+ neurons of the CSB are glutamatergic since they express EMX1 (arrowheads in A2). (B1–B2) In situ hybridizations for Rfx3 (red) combined with immunohistochemical staining for Calbindin (green) on coronal brain sections in WT mice at E16.5. Calbindin+ neurons of the CSB are glutamatergic since they express Emx1 (arrowheads in B2). (C1 and C2) Immunohistochemical staining for RFX3 in coronal sections from GAD67-GFP transgenic mice at E18.5 showing that RFX3 is not expressed by GABAergic interneurons. (D–H) In situ hybridizations for Rfx3 (red) combined with immunohistochemical staining for Nestin (D1–D2), GFAP (E1–E2 and F1–F2), SATB2 (G1–G2) or CTIP2 (H1–H2) (green) on coronal brain sections in WT mice at E14.5 (D1–D2), E16.5 (E1–E2) and E18.5 (F1–F2 to H1–H2). D2, E2, F2, G2 and H2 are high magnifications of D1, E1, F1 G1 and H1, respectively. (D1–D2 to F1–F2) Radial glial cells of the glial wedge (GW) labelled for Nestin and GFAP express high levels of Rfx3 (arrowheads). By contrast, astrocytes of the indusium griseum (IG) labelled for the same markers are devoided of Rfx3. (G1–G2 and H1–H2) At E18.5, Rfx3 is expressed by callosal pyramidal neurons (SATB2+) and by sub-cerebral projecting neurons (CTIP2+). (G2 and H2) High magnified views of the cortex showing that glutamatergic pyramidal neurons expressing the nuclear transcription factors SATB2 or CTIP2 contain cytosolic Rfx3 mRNAs (arrowheads). Bar = 435 µm in A1, G1, H1; 220 µm in B1, D1, E1; 110 µm in C1, C2; 60 µm in A2, B2; 40 µm in D2, E2, F1, F2, G2, H2. (TIF) [file pgen.1002606.s003.tif]

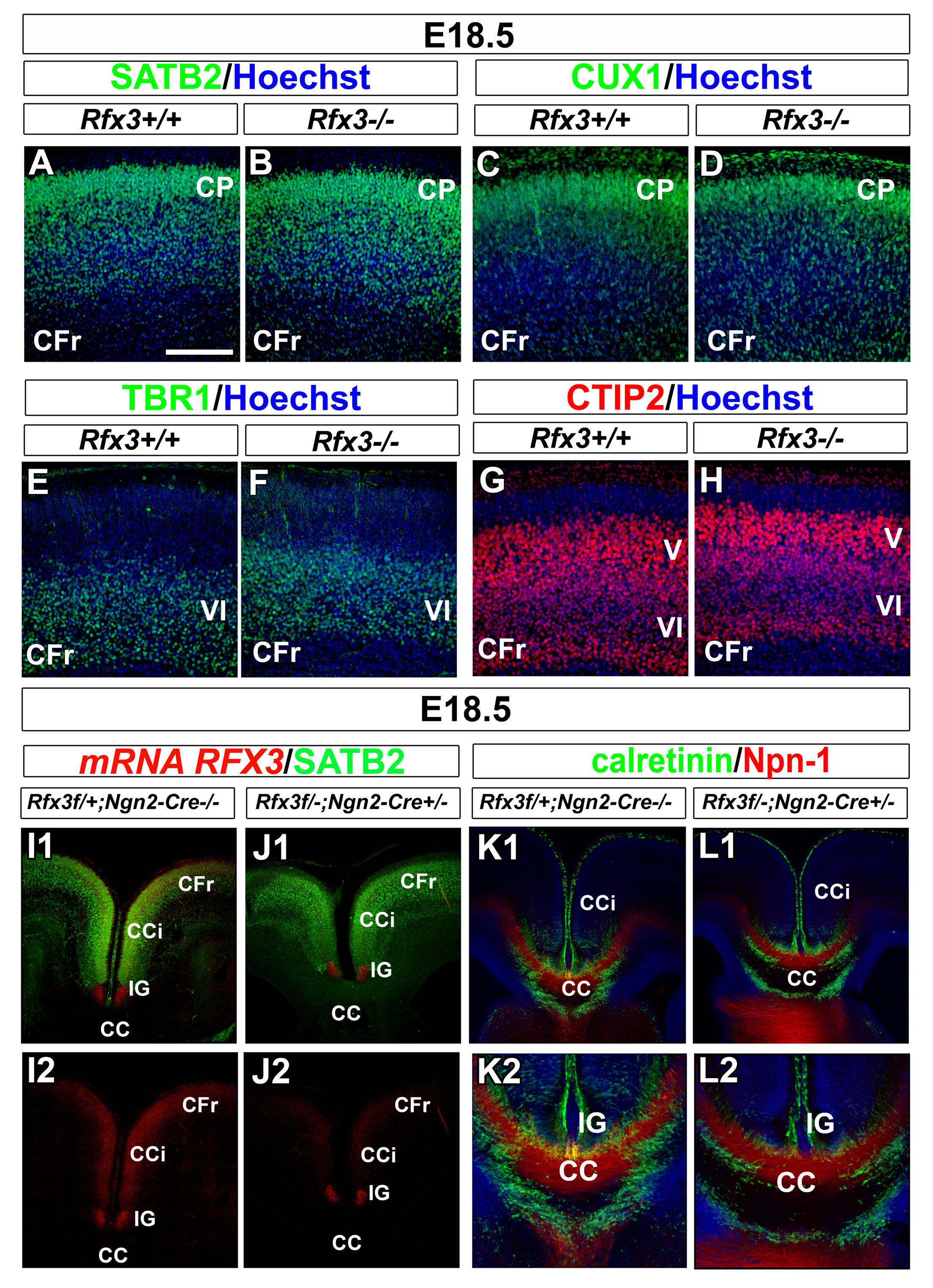

Supplement: Figure S4 — Rfx3 inactivation in cortical pyramidal neurons is not responsible for callosal axon guidance defects. (A–H) Single immunohistochemistry for SATB2 (A and B), for CUX1 (C and D), for TBR1 (E and F) or for CTIP2 (G and H) in coronal sections from E18.5 WT (A, C, E and G) and Rfx3 −/− (B, D, F and H) mice. SATB2+, CUX1+, TBR1+ and CTIP2+ cortical layers are not affected in the Rfx3−/ −. (I–J) In situ hybridizations for Rfx3 (in red) combined with immunohistochemical staining for SATB2 (in green) on coronal brain sections of control Rfx3 f/+ ;Ngn2-CreERtm−/− (I1–I2) and Rfx3 f/− ;Ngn2-CreERtm+/− (J1–J2) embryos at E18.5. (I1–I2) In control Rfx3 f/+ ;Ngn2-CreERtm−/− mice, Rfx3 is strongly expressed through the glutamatergic neurons of the cortex and of the indusium griseum (IG). (J1–J2) In Rfx3 f/− ;Ngn2-CreERtm+/− brains, Rfx3 hybridation signal is significantly decreased in all the cortical layers after induced recombination of Rfx3 floxed allele in Ngn2-derived cells. Rfx3 inactivation in the cortex does not affect the cortical distribution of SATB2+ callosal projecting neurons. (K–L) Double immunohistochemistry for calretinin and Npn-1 (K1–K2 and L1–L2) in coronal CC sections from E18.5 control Rfx3 f/+ ;Ngn2-CreERtm−/− (K1–K2) and Rfx3 f/− ;Ngn2-CreERtm+/− (L1–L2) mice. In brain sections of mice where Rfx3 is conditionally inactivated in Ngn2-derived cortical pyramidal neurons, callosal axons develop normally. Bar = 435 µm in I1, J1, I2, J2, K1, L1; 220 µm in K2, L2 and 110 µm in A, B, C, D, E, F, G, H. (TIF) [file pgen.1002606.s004.tif]

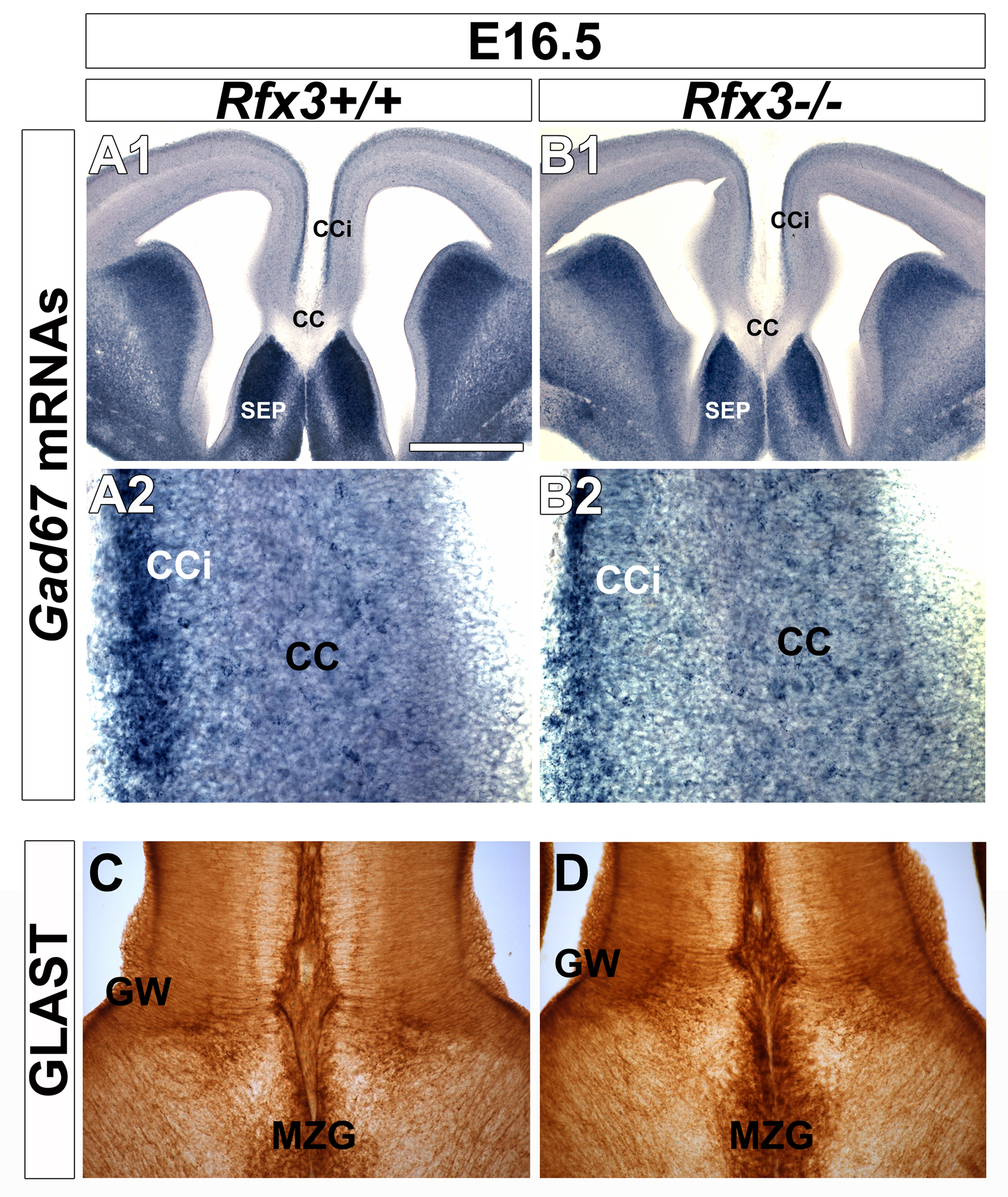

Supplement: Figure S5 — Localization of GABAergic interneurons in Rfx3 deficient mice. (A–B) In situ hybridization for Gad67 mRNAs on coronal CC slices of E16.5 Rfx3 deficient mice (B1–B2) in comparison with wild type (A1–A2). We observe a normal localization of Gad67 in telencephalon, and notably in the lateral CC region, of Rfx3 deficient mice (B1–B2) compared to wild type mice (A1–A2). (C–D) DAB staining for GLAST on coronal rostromedial slices from E16.5 WT (C) and Rfx3−/− (D) mice. We observe a normal localization and organization of guidepost glia of the midline zipper glia (MZG) and of the glial wedge (GW) of Rfx3 deficient mice (D) compared to wild type mice (C). Bar = 1200 µm in A1, B1, Bar = 300 µm in C, D, and 150 µm in A2, B2. (TIF) [file pgen.1002606.s005.tif]

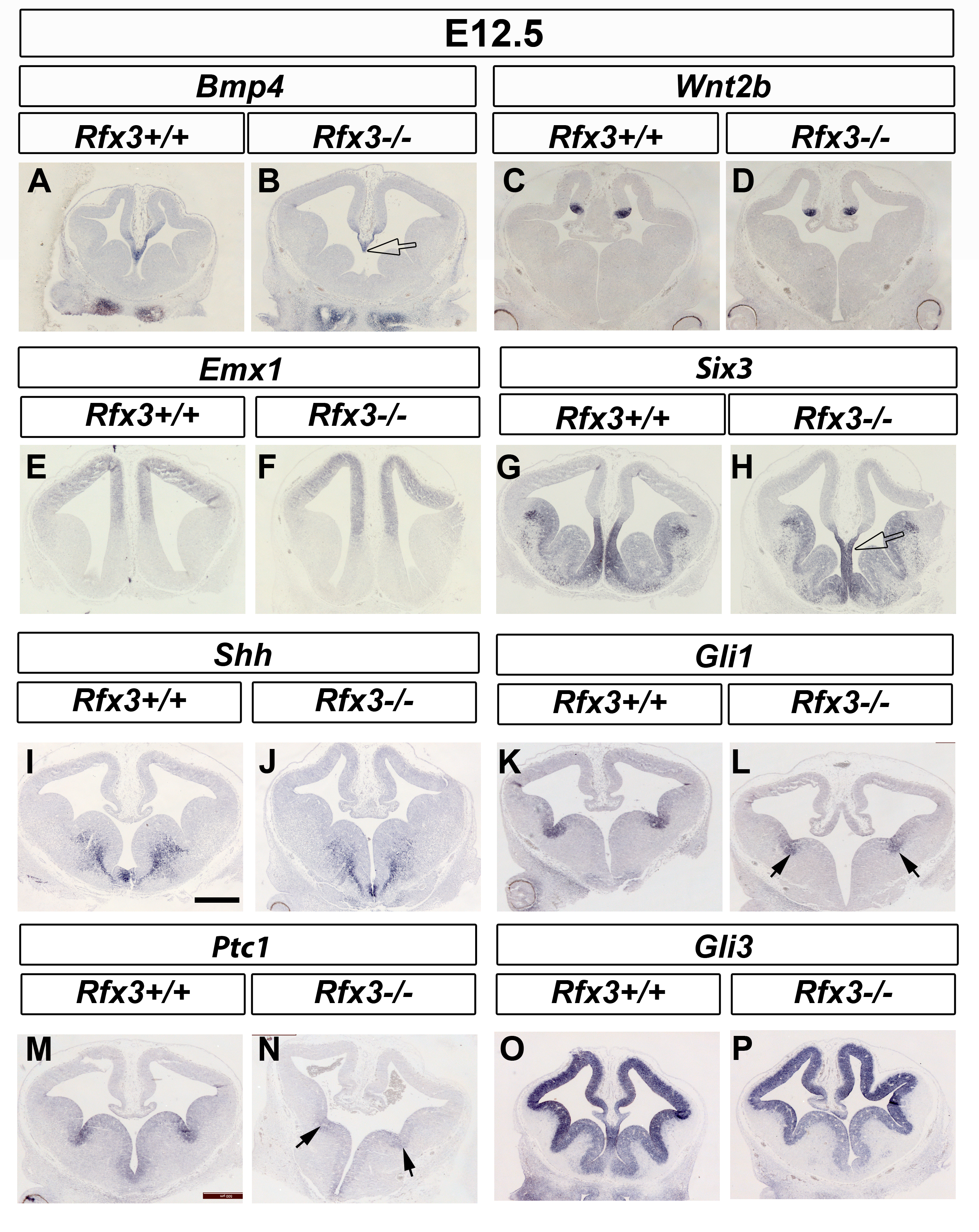

Supplement: Figure S6 — Expression of dorsal and ventral markers in Rfx3−/− brains. In situ hybridization for Shh (A and B), Gli1(C and D), ptc1(E and F), Gli3 (G and H), Bmp4 (I and J) and Wnt2b (K and L) mRNAs on coronal sections from E12.5 WT (A, C, E, G, I, K, M and O) and Rfx3−/− (B, D, F, H, J, L, N and P) mice. (A to D) All dorsal midline markers are normally expressed in the Rfx3 −/− embryos. (E to H) All commissural plate markers are normally expressed in the Rfx3 −/− embryos. (I to P) While Shh and Gli3 were properly specified, Ptc1 receptor and Gli1 are down-regulated (black arrows) in Rfx3−/−. Interestingly, the frontier region between the septum and the cortex is reduced in some mutants compare to WT (empty arrows). Bar = 500 µm in all. (TIF) [file pgen.1002606.s006.tif]

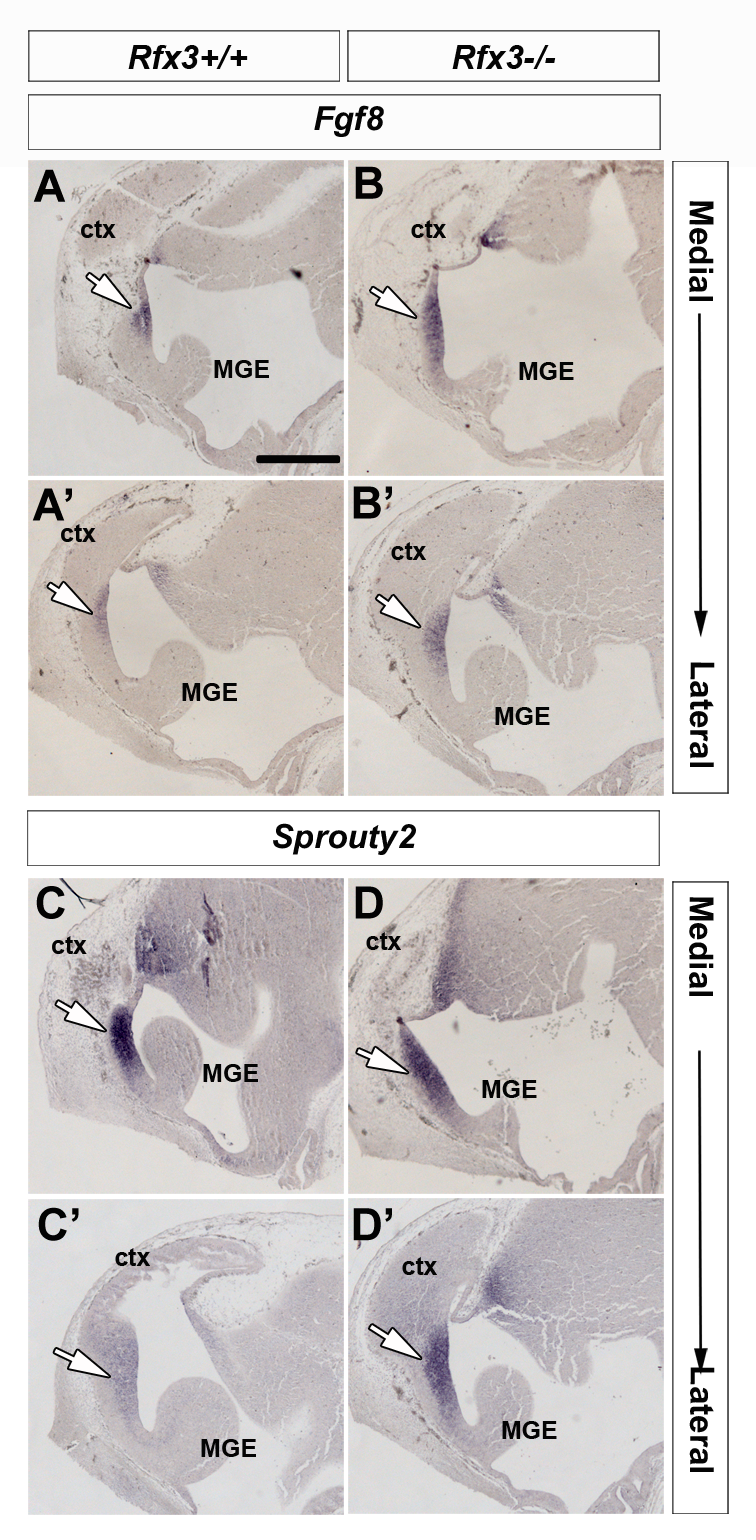

Supplement: Figure S7 — Enlargement of the Fgf8 expression domain in Rfx3−/− embryos. In situ hybridization for Fgf8 (A and B) and Sprouty2 (C and D) mRNAs on sagittal midline sections from E12.5 WT (A and C) and Rfx3−/− (B and D) embryos at the CSB. Fgf8 and Sprouty2 expression domains are expanded rostrally and laterally in the pallium of Rfx3−/− embryos (arrows). Bar = 500 µm in all figures. (TIF) [file pgen.1002606.s007.tif]
